# Three Ablation Techniques for Atrial Fibrillation during Concomitant Cardiac Surgery: A Systematic Review and Network Meta-Analysis

**DOI:** 10.3390/jcm12175716

**Published:** 2023-09-01

**Authors:** Dudy Arman Hanafy, Wahyu Prima Erdianto, Theresia Feline Husen, Ilona Nathania, Ananda Pipphali Vidya, Ruth Angelica, Widya Trianita Suwatri, Pasati Lintangella, Priscillia Prasetyo, Sugisman

**Affiliations:** 1 Division of Cardiothoracic and Vascular Surgery, Department of Surgery, Faculty of Medicine, University of Indonesia, Jl. Salemba Raya No. 6, Kenari, Jakarta 10430, Indonesia; hanafymedical@gmail.com (D.A.H.);; 2 Division of Adult Cardiac Surgery, Department of Surgery, National Cardiovascular Center, Harapan Kita, Jakarta 11420, Indonesia; wahyuprimae@gmail.com; 3 Faculty of Medicine, University of Indonesia, Pondok Cina, Beji, Depok 16424, Indonesia

**Keywords:** atrial fibrillation, bi-atrial ablation, left atrial ablation, pulmonary vein isolation

## Abstract

Atrial fibrillation (AF) ablation is a frequent procedure used in concomitant cardiac surgery. However, uncertainty still exists concerning the optimal extent of lesion sets. Hence, the objective of this study was to assess the results of various ablation techniques, aiming to offer a reference for clinical decision making. This review is listed in the prospective register of systematic reviews (PROSPERO) under ID CRD42023412785. A comprehensive search was conducted across eight databases (Scopus, Google Scholar, EBSCOHost, PubMed, Medline, Wiley, ProQuest, and Embase) up to 18 April 2023. Studies were critically appraised using the Cochrane Risk of Bias 2.0 for randomized control trials (RCTs) and the Newcastle Ottawa Scale adapted by the Agency for Healthcare Research and Quality (AHRQ) for cohort studies. Forest plots of pooled effect estimates and surface under the cumulative ranking (SUCRA) were used for the analysis. Our analysis included 39 studies and a total of 7207 patients. Both bi-atrial ablation (BAA) and left atrial ablation (LAA) showed similar efficacy in restoring sinus rhythm (SR; BAA (77.9%) > LAA (76.2%) > pulmonary vein isolation (PVI; 66.5%); LAA: OR = 1.08 (CI 0.94–1.23); PVI: OR = 1.36 (CI 1.08–1.70)). However, BAA had higher pacemaker implantation (LAA: OR = 0.51 (CI 0.37–0.71); PVI: OR = 0.52 (CI 0.31–0.86)) and reoperation rates (LAA: OR = 0.71 (CI 0.28–1.45); PVI: OR = 0.31 (CI 0.1–0.64)). PVI had the lowest efficacy in restoring SR and a similar complication rate to LAA, but had the shortest procedure time (Cross-clamp (Xc): PVI (93.38) > LAA (37.36) > BAA (13.89)); Cardiopulmonary bypass (CPB): PVI (93.93) > LAA (56.04) > BAA (0.03)). We suggest that LAA is the best surgical technique for AF ablation due to its comparable effectiveness in restoring SR, its lower rate of pacemaker requirement, and its lower reoperation rate compared to BAA. Furthermore, LAA ranks as the second-fastest procedure after PVI, with a similar CPB time.

## 1. Introduction

AF affects 37 million individuals worldwide, making it the most prevalent form of persistent cardiac arrhythmia. Its incidence has risen by 33% over the past two decades, with projections indicating a potential doubling in the next few decades [1]. Despite often being perceived as less harmful due to its primary impact being on the elderly, AF has a five-fold stroke risk and impairs the function of the cardiac system, leading to substantial morbidity, mortality, hospitalization, and a diminished quality of life [1,2]. In fact, AF’s five-year survival rate ranks 11th among the 25 most lethal malignancies in the United States [3]. These alarming findings highlight the urgent need for a proactive approach to AF treatment, including the utilization of ablation techniques.

Ablation techniques have revolutionized interventions for AF, surpassing alternative therapy options in terms of effectiveness [4]. Initially, electrical ablation using a catheter emerged as the favored treatment in the early 1980s. Subsequent advancements led to the development of radiofrequency ablation and cryoablation techniques, which proved to be safer and more effective. Guidelines have advocated for the incorporation of these ablations in concomitant coronary artery bypass grafting (CABG) and valve-related surgeries [3].

Different methods are employed for ablation procedures, including bi-atrial ablation (BAA), the Cox maze, Left atrial ablation (LAA), and Pulmonary vein isolation (PVI). BAA is performed by ablating the tissue around the pulmonary vein with circumferential lines, performed ≥1 cm from the pulmonary vein (PV) ostia, the inferior portion of the left inferior encircling lesion to the mitral annulus (mitral isthmus), the posterior intercaval line, the septal portion of the superior vena cava, the posteroseptal area of the ostium, the inferior vena cava, and the cavotricuspid isthmus—while LAA is performed by ablating the left atrium with lesions connected to the mitral valve and the tissue around the pulmonary veins only, continuing to the left atrial appendage; meanwhile, PVI is performed by ablating the tissue around the pulmonary veins with circumferential lines only. LAA and PVI serve as less-invasive alternatives to BAA, aiming to reduce bypass time, procedure-related harm, early mortality, and complications. However, the efficacy of these simplified procedures remains uncertain; hence, BAA continues to be considered the gold standard for AF therapy [4,5,6,7,8,9].

Despite BAA being considered the gold standard, conflicting findings from various studies have raised questions about the similarities and differences between BAA, LAA, and PVI in terms of ablation efficacy [3,10]. The limited availability of studies directly comparing the outcomes of these procedures and yielding inconclusive results has hindered definitive conclusions. Hence, our meta-analysis aims to directly compare the outcomes associated with the BAA, LAA, and PVI techniques, providing valuable guidance for clinical decision making.

## 2. Materials and Methods

This review was written following the guidelines provided in the Cochrane Handbook for Systematic Reviews of Interventions (version 6.3), and our findings were reported following the Preferred Reporting Items for Systematic Review and Meta-Analysis (PRISMA) guidelines. Our study was enlisted in the international prospective register of systematic reviews (PROSPERO) to ensure its novelty and adherence to research ethics, and it has been assigned the ID CRD42023412785.

### 2.1. Search Strategy

Five independent reviewers from Indonesia utilized internet browser conducted a thorough and extensive literature search using multiple electronic databases, including Scopus, PubMed, Cochrane, Google Scholar, EBSCOhost, Wiley, Medline, and ProQuest, up to 18 April 2023. The search strategy incorporated keywords such as atrial fibrillation, pulmonary vein isolation, left atrial ablation, and bi-atrial ablation, along with relevant synonyms. Whenever appropriate and feasible, advanced search techniques were employed to refine and narrow down the search results.

### 2.2. Study Eligibility Criteria

**Figure 1:**
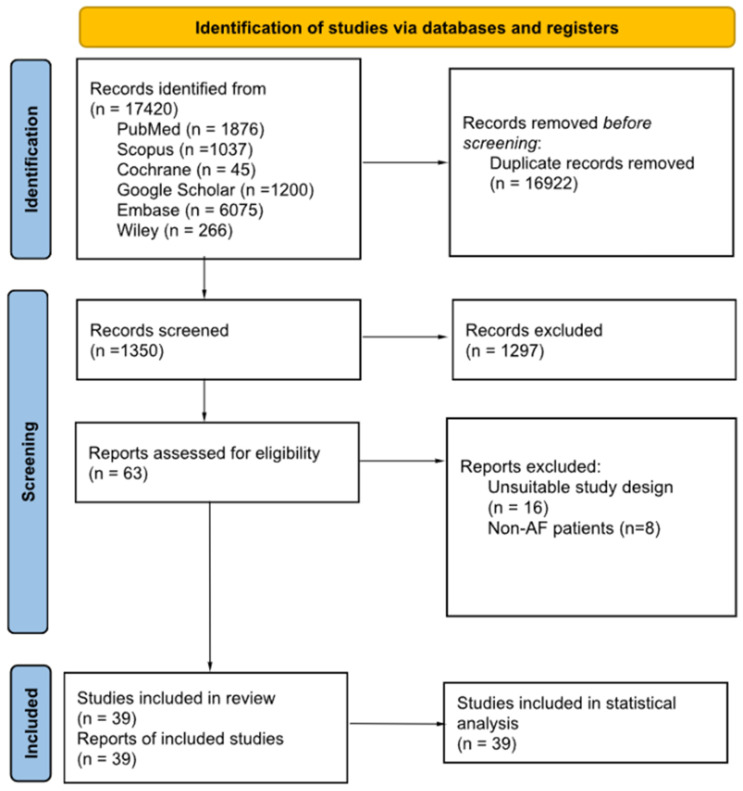
PRISMA.

The included studies underwent a thorough screening process based on specific eligibility criteria. The eligibility criteria encompassed: (1) observational studies or RCTs, (2) studies that compared the surgical ablation outcomes between PVI, LAA, BAA, or compared two out of these three techniques, (3) studies that involved patients diagnosed with atrial fibrillation, and (4) studies that reported both qualitative and quantitative outcomes. Conversely, we excluded (1) studies with unsuitable designs—such as letters to the editor, reviews, commentaries, and preclinical studies—and (2) studies that solely provided qualitative outcome data (Figure 1).

### 2.3. Data Extraction

Specific data elements from specified studies, including (1) the study ID (author, year); (2) study features, including the method and location of the study; (3) the sample details—encompassing the size, age, and technique types; and (4) the study outcomes, were extracted and organized in an Excel document. The results were further organized into three main sections: hospital stay, operative procedure, and postoperative outcomes. The hospital stay section encompassed overall hospital stays and admissions. The operative procedure section included data on Xc, CPB, and ablation times. The postoperative outcomes section was comprised of variables such as non-SR, the need for a pacemaker, reoperation, bleeding, reoperation for bleeding, cerebrovascular accidents (CVA), and overall mortality. Data extraction and quality checks for all the extracted data during the statistical analysis were carried out independently by five authors.

### 2.4. Risk of Bias in Assessments

The included studies underwent an appraisal using the Newcastle–Ottawa quality assessment scale for observational studies and the Cochrane Risk of Bias (RoB) 2.0 tool for RCTs. The Newcastle–Ottawa scale provided a qualitative evaluation, with scores converted to AHRQ standards for categorization [11,12]. The Cochrane Risk of Bias 2.0 tool assessed potential bias sources across five domains. To ensure consistency, five independent reviewers conducted the assessment, with blinded scoring. Consensus was reached through reviewer discussions. The results were presented succinctly and visualized using the RoB tool [13].

### 2.5. Statistical Analysis

We estimated the odds ratios (ORs) for dichotomous outcomes and mean differences (MDs) for continuous outcomes, with their 95% Cis, using conventional pairwise and network meta-analyses. All ORs and MDs from the included studies were interpreted using randomized pooled effects and displayed using forest plots. However, due to the presence of an open loop in the network, it was not possible to assess incoherency within the network. Therefore, heterogeneity was reported based on the random-effects model from the conventional pairwise forest plot analysis. In the event of potential heterogeneity among the studies, we employed the inverse variance DerSimonian–Laird random-effects model. This approach was chosen because we anticipated the presence of considerable heterogeneity across the studies. Heterogeneity was further assessed using the Cochrane threshold and expressed as the estimated effect (I^2^) statistics, with cut-off values of 0%, 25%, 50%, and 75% representing insignificant, low, moderate, and high heterogeneity, respectively [14].

For two-group outcomes, Review Manager ver. 5.4 (The Nordic Cochrane Center, The Cochrane Collaboration, Copenhagen) was used to produce a conventional pairwise meta-analysis. Meanwhile, in cases where the outcome involved more than two groups in the conventional meta-analysis, we conducted a Bayesian network meta-analysis (NMA) using MetaInsight V4.0.1 Beta [15]. 

We provided tables containing 95% CIs that provide statistically significant comparisons for each treatment (Table 1, Table 2 and Table 3). Additionally, we also provided figures that showed the number of trials, comparing each pair of treatments with the line width as well as the number of randomized participants with the node size. For the NMA, we utilized SUCRA to rank all techniques, with higher values indicating a lower complication rate and shorter procedure time—indicating the best treatment—and organized them into a table (Table 4). However, SUCRA is unable to rank improvement outcomes such as freedom from AF; it is only able to analyze complications such as non-sinus rhythms. Thus, we converted results related to rhythm improvement (freedom from AF) into percentages, as this is the most common units that are used. 

## 3. Results

### 3.1. Study Selection and Characteristics

A comprehensive search across six databases yielded a total of 17,420 studies, with details in supplementary materials Table S1 [6,9,10,16,17,18,19,20,21,22,23,24,25,26,27,28,29,30,31,32,33,34,35,36,37,38,39,40,41,42,43,44,45,46,47,48,49,50,51,52,53]. After removing duplicates and excluding ineligible studies, 63 studies were assessed against the eligibility criteria. Ultimately, 39 studies were included for the qualitative and quantitative analysis (Figure 1). Supplementary materials Table S2 lists the characteristics of these included studies.

This review included a total of 7207 patients from multiple studies. Ablation, which can be performed via a catheter or as a surgical procedure, is a treatment option for patients with atrial fibrillation. Our analysis specifically focused on the concurrent performance of ablation with cardiac surgery. The study considered three surgical ablation techniques: PVI, LAA, and BAA. Among the thirty-nine included studies, five directly compared all three intervention techniques. Additionally, seven studies compared PVI with BAA, two studies compared PVI with LAA, and twenty-four studies compared LAA with BAA.

### 3.2. Quality Assessment

**Figure 2:**
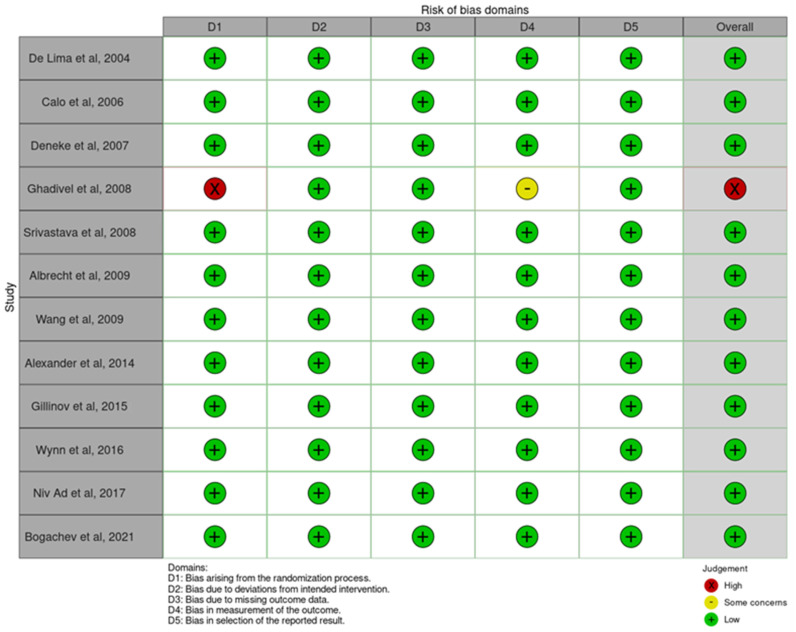
RoB Visualization [6,16,17,18,19,20,21,22,23,24,25,26].

All RCTs included in this study had a low risk of bias based on the Cochrane risk of bias 2.0, except for one clinical trial that had a high risk due to having a non-random selection process (Figure 2). The observational study was appraised using the Newcastle Ottawa Scale (NOS) [6,9,10,16,17,18,19,20,21,22,23,24,25,26,27,28,29,30,31,32,33,34,35,36,37,38,39,40,41,42,43,44,45,46,47,48,49,50,51,52,53]. All of these observational studies were rated as high quality with a low risk of bias (supplementary materials Table S6).

### 3.3. Postoperative Outcomes

**Figure 3:**
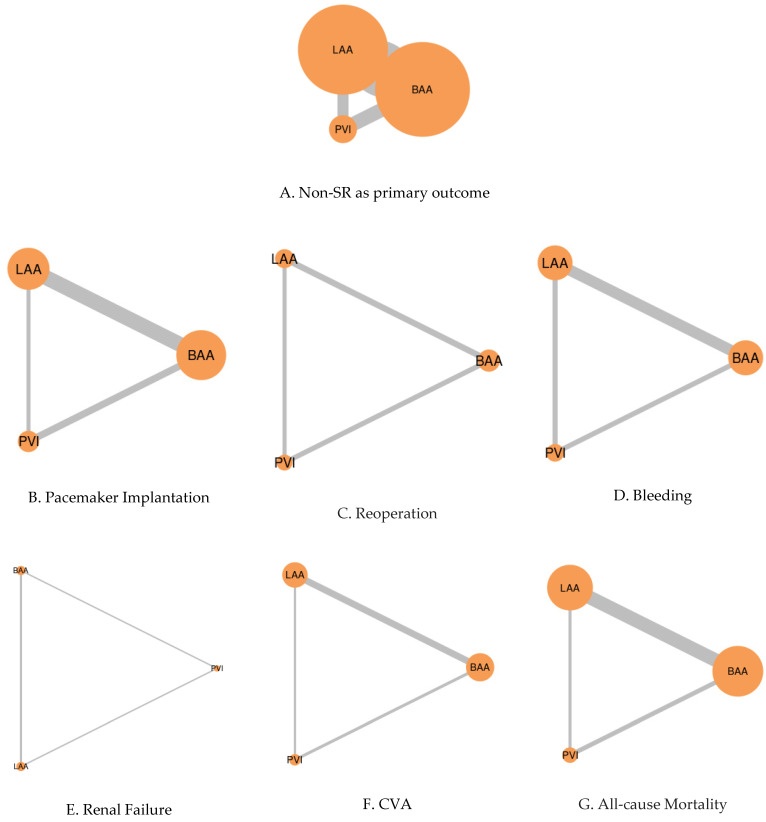
Network of comparisons between three ablation techniques with regards to postoperative outcomes. The width of the lines is proportional to the number of trials comparing each pair of treatments. Meanwhile, the size of the nodes is proportional to the number of randomized participants.

This study assessed six domains of postoperative outcomes, which were non-sinus rhythm, pacemaker requirement, reoperation, bleeding, cerebrovascular accidents (CVA), and overall mortality (supplementary materials Table S3). The results showed that BAA (92.28) and LAA (56.31) had similar rates of non-sinus rhythms, while PVI (1.41) had the highest rate among the three techniques. The SUCRA rank was converted to simplified percentages, indicating that BAA (77.9%) had the highest ranking—followed closely by LAA (76.2%) and then PVI (66.5%). Regarding the need for a pacemaker (temporary and permanent), BAA (0.31) had the highest requirement compared to the other two techniques, which showed similar outcomes (LAA: 77.25; PVI: 72.45). For reoperation, PVI (98.31) and LAA (43.56) exhibited comparable rates, while BAA (8.13) had the highest rate among the three techniques. Complication rates—including bleeding, renal failure, CVA, and mortality—were similar across all three techniques (Table 1 and Table 4; Figure 3; supplementary materials Figure S1). The level of heterogeneity varied from insignificant to low across these analyses.

### 3.4. Operative Procedure Time

**Figure 4:**
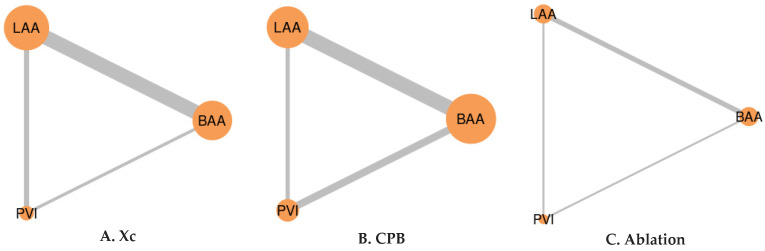
Network of comparisons between the three ablation techniques’ procedure times. The width of the lines is proportional to the number of trials comparing each pair of treatments. Meanwhile, the size of the nodes is proportional to the number of randomized participants.

Cross-clamp time (Xc) was assessed in 16 studies, as detailed in supplementary materials Table S4. Regarding Xc time, PVI ranked highest based on the SUCRA analysis—showing the shortest time (93.38)—followed by LAA (37.36) and BAA (13.89), although LAA and BAA were comparable. Meanwhile, for the cardiopulmonary bypass (CPB) times—which were observed in 12 studies—BAA (0.03) had the longest time, followed by comparable times for LAA (56.04) and PVI (93.93). However, when considering ablation time—as reported in seven studies—only PVI demonstrated a significantly shorter time (99.18), while there was no significant difference between LAA (49.08) and BAA (1.74; Table 2 and Table 4; Figure 4; supplementary materials Figure S2). The level of heterogeneity ranged from insignificant to high across these analyses.

### 3.5. Hospital Admissions and Stays

**Figure 5:**
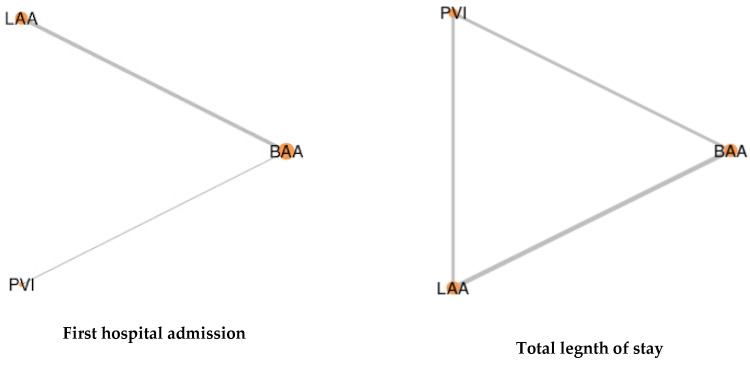
Network of comparisons between the three ablation techniques effect with regards to hospital admissions and stays. The width of the lines is proportional to the number of trials comparing each pair of treatments. Meanwhile, the size of the nodes is proportional to the number of randomized participants.

Four studies included in this analysis reported initial hospital admissions following surgical ablation (supplementary materials Table S5). Additionally, three studies provided information on the overall length of patient stays. The findings indicate that all three surgical ablation techniques yielded similar results when it came to hospital admissions and length of stay (Table 3 and Table 4; Figure 5 and Figure S3). The level of heterogeneity varied from insignificant to low across these analyses.

## 4. Discussion

As previously mentioned, surgical ablation has proven to be the most effective treatment for AF. However, determining the optimal lesion sets to use remains inconclusive. Some opinions suggest that a broader range of ablation techniques may result in better rhythm outcomes, but this carries higher surgical risks. A previous meta-analysis by Guo et al. reported that BAA yielded the best results for SR, followed by LAA and then PVI, but the differences were not statistically significant [3]. However, our meta-analysis presents different findings, where BAA and LAA show similar results and PVI demonstrates inferior outcomes.

This comprehensive meta-analysis aimed to assess the efficacy of three distinct procedures—BAA, LAA, and PVI—for the treatment of AF. By analyzing data from 18 RCTs and 21 observational studies involving 7207 patients, we categorized the outcomes into three main sections: operative procedure, postoperative outcomes, and hospital admissions and stay.

In terms of the operative procedure, our findings revealed that both PVI and LAA techniques exhibited shorter cardiopulmonary bypass (CPB) and cross-clamp (Xc) times compared to BAA. Specifically, the ranking order for Xc time was as follows: PVI (93.38) > LAA (37.36) > BAA (13.89); and for CPB time: PVI (93.93) > LAA (56.04) > BAA (0.03). These results align with the study conducted by Stulak et al., which demonstrated similar significant findings among 1540 patients who underwent surgical ablation for AF [19]. The implementation of advanced operative technologies and myocardial surgery protection strategies, including the use of bipolar radiofrequency energy, has contributed to these favorable outcomes. Importantly, despite the slightly longer Xc and CPB times, the utilization of these modern strategies does not increase perioperative risks [1,6].

Moving on to the evaluation of postoperative outcomes, our analysis encompasses six key domains: non-SR, the need for a pacemaker, reoperation, bleeding, reoperation for bleeding, cerebrovascular accidents (CVA), and overall mortality. The results indicated that both BAA and LAA techniques exhibited higher rates of freedom from AF when compared to PVI (BAA 77.9% > LAA 76.2% > PVI 66.5%). These findings are consistent with the network meta-analysis conducted by Guo et al. (BAA 88.97% > LAA 74.91% > PVI. 36.12%) and the study by Gauzebroek et al. (BAA 60% vs. LAA 59% vs. PVI 33%) [3,30]. Although our findings are consistent with previous meta-analyses, the difference between PVI and BAA or LAA is not as substantial. Notably, a systematic review by Sef et al. reported no significant differences in freedom from AF between PVI and BAA. They mentioned the technical complexity of BAA, the effective application of lesions, increased cross-clamp time, and higher permanent pacemaker implantation as limiting factors for BAA. PVI, on the other hand, had advantages in high-risk patients due to shorter operation times and restricted ablation lesion sets [53]. These results differ from our findings, which stated that BAA is more effective than PVI—although with a similar efficacy to LAA. 

While BAA showed a better rate of restoring SR, it is worth noting that it necessitates the highest rate of pacemaker implantation and reoperation compared to the other techniques [3]. This finding aligns with the meta-analysis by Cappabianca et al., which demonstrated a higher incidence of permanent pacemaker (PPM) implantation and reopening for bleeding in the BAA group [54]. These outcomes may be attributed to a higher prevalence of sinoatrial node dysfunction in the BAA group. In terms of other complications, mortality, and hospital stays, our study revealed no significant differences among the three surgical ablation techniques, which aligns with previous findings [3,49,55]. This concurrence further strengthens the reliability of our results in these aspects.

Overall, our meta-analysis provides valuable insights into the efficacy of BAA, LAA, and PVI for the treatment of AF. The results contribute to the existing body of knowledge and can guide clinical decision making in selecting the most appropriate procedure based on individual patient characteristics.

### Strength and Limitations

Based on our knowledge, it is worth noting that only a limited number of studies have directly assessed these three surgical ablation techniques. Moreover, our study boasts a substantial sample size of patients, and a significant portion of the results obtained hold promising significance—thereby providing representative and reliable data. Our analysis also revealed a low risk of bias for this meta-analysis, as assessed using the Newcastle–Ottawa scale and the Cochrane Risk of Bias 2.0 tool. However, it is important to acknowledge that despite these strengths, our study does have certain limitations. One such limitation is the presence of high heterogeneity in some subgroup results, which may be influenced by various factors across different studies, such as the types of studies being included (observational and RCTs), the variation in the concomitant surgeries performed, and the atrial fibrillation types.

## 5. Conclusions

We suggest that LAA is the best surgical ablation technique for AF; this technique demonstrated comparable effectiveness in restoring SR, with a lower rate of pacemaker requirement and reoperation rate compared to BAA. Additionally, LAA is the second-fastest procedure after PVI, with a similar CPB time.

## 6. Patents

This section is not mandatory but may be added if there are patents resulting from the work reported in this manuscript.

## Figures and Tables

**Table 1:** Bayesian network meta-analysis of postoperative outcomes.

Non-SR	BAA	LAA	PVI
BAA	BAA	1.08(0.94, 1.23)	1.36 (1.08, 1.7) *
LAA	0.93 (0.81, 1.07)	LAA	1.26 (1.01, 1.6) *
PVI	0.74 (0.59, 0.93) *	0.79 (0.63, 0.99) *	PVI
**Pacemaker Implantation**	BAA	LAA	PVI
BAA	BAA	0.52 (0.37, 0.71) *	0.48 (0.3, 0.8) *
LAA	1.93 (1.4, 2.69) *	LAA	0.93 (0.56, 1.62)
PVI	2.08 (1.26, 3.38) *	1.08 (0.62, 1.8)	PVI
**Reoperation**	BAA	LAA	PVI
BAA	BAA	0.71 (0.28, 1.45)	0.31 (0.1, 0.64) *
LAA	1.4 (0.69, 3.61)	LAA	0.43 (0.16, 1.05)
PVI	3.26 (1.56, 9.53) *	2.34 (0.95, 6.29)	PVI
**Bleeding**	BAA	LAA	PVI
BAA	BAA	0.58 (0.22, 1.5)	0.74 (0.18, 2.43)
LAA	1.71 (0.67, 4.57)	LAA	1.26 (0.32, 4.24)
PVI	1.35 (0.41, 5.54)	0.79 (0.24, 3.16)	PVI
**Renal Failure**	BAA	LAA	PVI
BAA	BAA	0.58 (0.22, 1.48)	0.75 (0.19, 2.36)
LAA	1.71 (0.68, 4.51)	LAA	1.27 (0.32, 4.15)
PVI	1.34 (0.42, 5.35)	0.79 (0.24, 3.11)	PVI
**CVA**	BAA	LAA	PVI
BAA	BAA	0.76 (0.14, 3.18)	0.89 (0.1, 6.43)
LAA	1.32 (0.31, 7.05)	LAA	1.17 (0.13, 10.56)
PVI	1.12 (0.16, 9.68)	0.86 (0.09, 7.45)	PVI
**All-cause Mortality**	BAA	LAA	PVI
BAA	BAA	1.06 (0.59, 1.78)	1.29 (0.52, 2.69)
LAA	0.94 (0.56, 1.7)	LAA	1.22 (0.5, 2.68)
PVI	0.78 (0.37, 1.93)	0.82 (0.37, 1.99)	PVI

Data are in OR (95% CI). As long as the ORs did not pass 1 (<1 or >1), they were considered statistically significant. Significance is marked by *.

**Table 2:** Bayesian network meta-analysis of operative procedure time.

Xc	BAA	LAA	PVI
BAA	BAA	−2.35 (−10.46, 5.43)	−16.2 (−29.78, −3.2)
LAA	2.35 (−5.43, 10.46)	LAA	−13.86 (−26.34, −1.45)
PVI	16.2 (3.2, 29.78) *	13.86 (1.45, 26.34) *	PVI
**CPB**	BAA	LAA	PVI
BAA	BAA	−18.91 (−29.25, −8.77) *	−27.39 (−41.04, −13.95) *
LAA	18.91 (8.77, 29.35) *	LAA	−8.48 (−23.14, 6.23)
PVI	27.39 (13.95, 41.04) *	8.48 (−6.23, 23.14)	PVI
**Ablation**	BAA	LAA	PVI
BAA	BAA	−7.4 (−15.56, 0.65)	−20.39 (−31.85, −9.44) *
LAA	7.4 (−0.65, 15.56)	LAA	−13 (−24.32, −2.03) *
PVI	20.39 (9.44, 31.85) *	13 (2.03, 24.32) *	PVI

Data are in MDs (95% CI; minutes). As long as the MDs did not pass 1 (<1 or >1), they were considered statistically significant. Significance is marked by *.

**Table 3:** Bayesian network meta-analysis of hospital admissions and stay.

Admission	BAA	LAA	PVI
BAA	BAA	1.8 (0.36, 7.16)	0.84 (0.08, 9.1)
LAA	0.55 (0.14, 2.76)	LAA	0.47 (0.03, 8.47)
PVI	1.18 (0.11, 13.14)	2.14 (0.12, 32.16)	PVI
Length of stay	BAA	LAA	PVI
BAA	BAA	−1.5 (−4.07, 1.1)	−1.83 (−5.56, 1.81)
LAA	1.5 (−1.1, 4.07)	LAA	0.33 (−3.52, 2.79)
PVI	1.83 (−1.81, 5.56)	0.33 (−2.79, 3.52)	PVI

Data are in MDs (95% CI; days). As long as the MDs did not pass 1 (<1 or >1), they were considered statistically significant.

**Table 4:** Ranking of SUCRA values of all parameters.

Treatment	Non-SR	Pacemaker Implantation	Reoperation	Bleeding	Renal Failure	Cerebrovascular Accidents	All-cause Mortality	Xc Time	CPB Time	Ablation Time	Hospital Admissions	Length of Stay
BAA	1 (92.28)	2 (0.31)	1 (8.13)	1 (20.05)	1 (50.06)	1 (40.39)	1 (64.87)	2 (13.89)	2 (0.03)	2 (1.74)	1 (61.60)	1 (13.29)
LAA	1 (56.31)	1 (77.25)	1 (43.56)	1 (77.13)	1 (75.89)	1 (60.08)	1 (54.98)	2 (37.36)	1 (56.04)	2 (49.08)	1 (23.93)	1 (65.23)
PVI	2 (1.41)	1 (72.45)	1 (98.31)	1 (52.81)	1 (24.05)	1 (49.53)	1 (30.15)	1 (98.76)	1 (93.93)	1 (99.18)	1 (64.46)	1 (71.48)

Legend: 1 = Best; 2 = Second; 3 = Third (SUCRA values). Same order means that there was no significant difference between the two/three treatments.

## Data Availability

No new data were created or analyzed in this study. Data sharing is not applicable to this article.

## Supplementary Materials

The following supporting information can be downloaded at: https://www.mdpi.com/article/10.3390/jcm12175716/s1, Table S1. Results of Databases Search. Table S2. Patients Baseline Characteristics. Table S3. Postoperative Outcomes. Table S4. Operative Procedure Time. Table S5. Hospital Admissions and Stay. Figure S1. Overall postoperative outcomes. Figure S2. Overall operative procedure. Figure S3. Hospital admission and stay. Table S6. Critical Appraisal.
