# Supplementary material for: Three Ablation Techniques for Atrial Fibrillation during Concomitant Cardiac Surgery: A Systematic Review and Network Meta-Analysis
Source: J Clin Med. 2023 Sep 1;12(17):5716. doi: 10.3390/jcm12175716 (PMC10488688; doi:10.3390/jcm12175716)
Supplement: Supplementary file 1 [file jcm-12-05716-s001.zip › jcm-2519251-supplementary.pdf]

Table S1. Results of Databases Search

Database	Keyword(s)	Hits
Pubmed	((Atrial fibrillation) OR (AF) OR (fibrillation of atrial)) AND ((Pulmonary vein isolation) OR (PVI) OR (pulmonary vein ablation)) AND ((Left atrial maze) OR (left atrial ablation) OR (cox maze ablation) OR (biatrial ablation))	1,876
Google Scholar	((Atrial fibrillation) OR (AF) OR (fibrillation of atrial)) AND ((Pulmonary vein isolation) OR (PVI) OR (pulmonary vein ablation)) AND ((Left atrial maze) OR (left atrial ablation) OR (cox maze ablation) OR (biatrial ablation))	1,200
Cochrane	((Atrial fibrillation) OR (AF) OR (fibrillation of atrial)) AND ((Pulmonary vein isolation) OR (PVI) OR (pulmonary vein ablation)) AND ((Left atrial maze) OR (left atrial ablation) OR (cox maze ablation) OR (biatrial ablation))	45
Scopus	((Atrial fibrillation) OR (AF) OR (fibrillation of atrial)) AND ((Pulmonary vein isolation) OR (PVI) OR (pulmonary vein ablation)) AND ((Left atrial maze) OR (left atrial ablation) OR (cox maze ablation) OR (biatrial ablation))	1,037
Wiley	((Atrial fibrillation) OR (AF) OR (fibrillation of atrial)) AND ((Pulmonary vein isolation) OR (PVI) OR (pulmonary vein ablation)) AND ((Left atrial maze) OR (left atrial ablation) OR (cox maze ablation) OR (biatrial ablation))	266
Embase	((Atrial fibrillation) OR (AF) OR (fibrillation of atrial)) AND ((Pulmonary vein isolation) OR (PVI) OR (pulmonary vein ablation)) AND ((Left atrial maze) OR (left atrial ablation) OR (cox maze ablation) OR (biatrial ablation))	6,075
Medline	((Atrial fibrillation) OR (AF) OR (fibrillation of atrial)) AND ((Pulmonary vein isolation) OR (PVI) OR (pulmonary vein ablation)) AND ((Left atrial maze) OR (left atrial ablation) OR (cox maze ablation) OR (biatrial ablation))	130

ProQuest	((Atrial fibrillation) OR (AF) OR (fibrillation of atrial)) AND ((Pulmonary vein isolation) OR (PVI) OR (pulmonary vein ablation)) AND ((Left atrial maze) OR (left atrial ablation) OR (cox maze ablation) OR (biatrial ablation))	6791
	**TOTAL**	**17,420**
	**ABSTRACT SCREENED**	**53**
	**INCLUDED**	**39**

Table S2. Patients Baseline Characteristics

No	Author, year	Study Design	Location	Sample Size	Age (Range/ Mean±SD)			Gender (M/F)			Study period	Follow-up (median (IQR)/ IQR/ Mean±SD/months mentioned)) (months)	Type of ablation	Lesion set	Concomitant Cardiac Surgery	Intervention (BAA vs PVI)			Types of Atrial Fibrilation (AF)
					BAA	LAA	PVI	BAA	LAA	PVI						BAA	LAA	PVI	
1	Hald et al, 2021	Cohort retrospective	Denmark	446	68.3 ± 9.2	N/A	70.0 ± 8.1	88/21	N/A	267/70	2004-2019	3-12	Radiofrequency	LAA, PVI, RAA	MVS, TVS, AVS, CABG	109	N/A	337	Paroxysmal and/or persistent AF
2	Albrecht et al, 2009	Randomized controlled clinical trial	Brazil	40	51.7 ± 12.4	N/A	55.1 ± 9.2	6/14	N/A	6/14	1999-2004	6-24	Cut and sew, radiofrequency	LAA,RAA,PVI	MVS	20	N/A	20	Paroxysmal AF
3	Srivastava et al, 2008	Randomized controlled clinical trial	India	120	37.11 ± 11.12	36.03 ± 7.99	40.95 ± 11.41	19/21	22/18	20/20	2000-2005	44	Radiofrequency, cyroablation	LAA, PVI, RAA	MVS, TVS	40	40	40	N/A
4	Stulak et al, 2014	Cohort retrospective	United States	893	514	110	269	N/A	N/A	N/A	1993-2011	33	Cut and sew, radiofrequency, Cyroablation	LAA, PVI, RAA	Repair of congenital heart disease : 351 (30.0) Repair of adult-acquired disease :1,189 patients (70.0)	514	110	269	Paroxysmal and/or persistent AF
5	Onorati et al, 2011	Observational prospective	Italy	141	64 ± 9	65 ± 8	N/A	79/30	18/14	N/A	2003-2008	1-60	Radiofrequency	LAA, RAA	MVS, AVS	109	32	N/A	Persistent AF
6	Alexander et al, 2018	Randomized controlled clinical trial	Russia	52	N/A	58.2 ± 7.9	56.5 ±6.9	N/A	11/14	11/16	2009-2011	18.6 ± 2.1	Radiofrequency	LAA,RAA,PVI	MVS, Left atrium thrombectomy,TVS, CABG	N/A	25	27	Paroxysmal AF
7	Gauzebroek et al, 2008	Cohort retrospective	Netherlands	65	63.3 ± 7.9	61.1 ± 10.3	59.6 ± 13.5	20/5	10/18	7/5	1999-2005	3.1–81.2	Cut and sew, radiofrequency, Cyroablation	PVI, LAA, RAA	CABG, TVS, MVS, AVS	25	28	12	Paroxysomal and/or Persistent and/or Permanent AF
8	Ghadivel et al, 2008	Clinical Trial	Iran	90	49 ± 12.6	50 ± 12.4	N/A	7/18	24/41	N/A	2004-2005	1-12	Cyroablation	PVI, LAA, RAA	MVS, AVS	25	65	N/A	Persisten AF with mitral valve
9	Gillinov et al, 2015	Randomized Controlled Trial	Canada	106	N/A	N/A	N/A	N/A	N/A	N/A	2010-2013	6,12	Cryoablation	PVI, LAA, RAA	CABG, TVS, MVS, AVS	47	N/A	59	Persistent or longstanding persistent AF who required mitral valve surgery
10	Yildirim et al, 2022	A Single Centered Retrospective Study	Germany	268	N/A	66.0 ± 8.6	67.4 ± 6.5	N/A	51/35	115/67	2003 - 2016	3, 12	Radiofrequency and cryoablation	LAA, PVI, MI	AVR, CABG	N/A	86	182	Paroxysmal AF
11	Wynn et al, 2016	Multicenter Randomized Contriolled Trial	England	124	61.9 ± 11.4	N/A	61.8 ± 9.7	39/24	N/A	45/16	2011-2013	3-12	Radiofrequency	PVI, LAA, RAA	lone surgery	63	N/A	61	Persistent or Sustained Paroxysomal AF
12	Wang et al, 2009	Prospective, open, and randomized trial with parallel groups	China	299	53.4 ± 10.8	54.2 ± 10.1	N/A	54/96	62/87	N/A	2004-2007	3-12	Radiofrequency	LAA,RAA,CS	CABG, TVS, MVS, AVS, Left atrial thrombectomy and folding	150	149	N/A	Persistent and/or Paroxysomal AF
13	Calo et al, 2006	Prospective, open, and randomized trial with parallel groups	Italy	80	57.9 ± 8.9	59.2 ± 9.	N/A	26/13	26/15	N/A	N/A	14 ± 5	Radiofrequency	MI, PVI, CS, cavotricuspid isthmus	MVS,TVS	39	41	N/A	Persistent or Permanent AF
14	Henn et al, 2015	Cohort retrospective	United States	109	N/A	N/A	N/A	N/A	N/A	N/A	2002-2014	12-60	Radiofrequency	PVI, LAA, RAA	MVR, DVR, TVP	61	48	N/A	Persistent or Paroxysomal or Non paroxysomal AF
15	Albage et al, 2011	Prospective clinical studies	Sweden	115	64.9 ± 10.4	66.9 ± 6.7	N/A	34/10	54/17	N/A	2005-2010	1-12	Cryoablation	PVI, PW, MI, LAA, Maze III	AVS, CABG, MVS, TVS	44	71	N/A	Paroxysmal, persistent, or permanent AF
16	McCarthy et al, 2010	Prospective clinical studies	United States	316	68.7 ± 10.3	66.8 ± 12.1	73.0 ± 10.7	42/88	13/22	41/9	2004-2008	5-24	Cut and sew, radiofrequency, Cyroablation	PVI, LAA, RAA, TC	AVR, MVR, TVP, CABG	91	175	50	Paroxysmal, persistent, or permanent AF
17	Guden et al, 2003	Cohort prospective	Turkey	105	52 ± 11	54 ± 9	N/A	14/34	23/34	N/A	2001	2-24	Radiofrequency	PVI, PW, MI, LAA, Maze III	AVS,CABG, MVS, TVS	48	57	N/A	Permanent or persistent AF
18	Pecha et al, 2014a	Nonrandomized retrospective study, Cohort	Germany	594	59 ± 28	68 ± 12	N/A	54	116	N/A	2003–2012	1	Radiofrequency and cryoablation	LVI, LAA, BLI, CI, RAA, TC	AVS, MVS, TVS, CABG	131	463	N/A	Paroxysmal, persistent, or permanent AF
19	Gualis et al, 2016	nonrandomized retrospective study, Cohort	Spain	150	65.1 ± 10.2	71.6 ± 6.8	N/A	29/38	39/44	N/A	2006–2011	36	Cryoablation	PVI, LAA, RAA, TC, CS, WG	AVS, MVS, TVS	67	83	N/A	Paroxysmal, persistent, or permanent AF
20	Deneke et al, 2009	Nonrandomized retrospective study, Cohort	Germany	130	53.4 ± 10.8	54.2 ± 10.1	N/A	54/10	62/4	N/A	1998-2004	51 ± 13	Radiofrequency	PVI, PW, MI, LAA, Maze III	MVS, AVS, CABG	64	66	N/A	Paroxysmal, persistent, or permanent AF
21	Pecha et al, 2014b	nonrandomized retrospective study, Cohort	Germany	132	70.5 ± 7.3	70.1 ± 7.5	N/A	45/11	40/26	N/A	2008–2011	12	Radiofrequency and cryoablation	LVI, LAA, CI, RAA, TC	AVS,CABG, MVS, TVS	66	66	N/A	Paroxysmal, persistent, or permanent AF
22	Soni et al, 2013	nonrandomized retrospective study, Cohort	United States	305	59 ± 28	68 ± 12	N/A	54/37	116/98	N/A	2007–2011	12	Radiofrequency, microwave and cryoablation	PVI, PW, MI, LAA, MM	AVS, MVS, CABG, TVS	91	214	N/A	Paroxysmal, persistent, or permanent AF
23	Kim et al, 2011	nonrandomized retrospective study, Cohort	South Korea	284	56.3 ± 12.0	52.1 ± 11.9	N/A	75/124	47/38	N/A	2006-2009	26.0 ± 13.3	Cryoablation	PVI, PW, MI, LAA, MM, CS	AVS,CABG, MVS, TVS	199	85	N/A	Paroxysmal, persistent, or permanent AF
24	Breda et al, 2011	nonrandomized retrospective study, Cohort	Brazil	30	60.0 ± 8.07	46.3 ± 9.54	N/A	9/6	5/10	N/A	2003-2009	12.16 ± 10.89	Radiofrequency	PVI, PW, MI, LAA, MM	MVS	15	15	N/A	Permanent or persistent AF
25	Deneke et al, 2007	Randomized Contriolled Trial	Germany	222	N/A	N/A	N/A	N/A	N/A	N/A	1997–2005	21	Radiofrequency	PVI, MV, LAA, RAA, MM	AVS,CABG, MVS	106	116	N/A	Paroxysmal, persistent, or permanent AF
26	Ryan et al, 2004	Non-randomized retrospective study, Cohort	United States	58	N/A	N/A	N/A	N/A	N/A	N/A	1996-2003	19.8 ± 25	Cut and sew, radiofrequency, cyroablation	PVI, PW, MI, LAA, Maze III	AVS,CABG, MVS, TVS	36	7	15	Paroxysmal, persistent, or permanent AF
27	Takami et al, 2019	Non-randomized retrospective study, Cohort	Japan	50	54.7 ± 8.8	58.3 ± 8.7	N/A	11/19	9/11	N/A	N/A	8-51	Cut and sew, radiofrequency, cyroablation	PVI, PW, MI, LAA, CTI, Maze III	AVS,CABG, MVS, TVS	30	20	N/A	Paroxysmal, persistent, or permanent AF
28	Takasaki et al, 2012	Non-randomized retrospective study, Cohort	Japan	75	59.6 ± 9.5	N/A	56.2 ± 7.3	17/18	N/A	19/21	1999-2004	>60	Cryoablation	PVI, LAA, RAA	AVS,CABG, MVS, TVS	35	N/A	40	Paroxysmal or persisten AF
29	Charitos et al, 2015	Non-randomized retrospective study, Cohort	Germany	51	N/A	N/A	N/A	N/A	N/A	N/A	N/A	21 ± 13.92	Cryoablation	PVI, LAA, RAA	AVS,CABG, MVS, TVS	29	22	N/A	Paroxysmal, persistent,or long-lasting persistent
30	Churyla et al, 2017	Non-randomized retrospective study, Cohort	United States	294	68.0 ± 11.6	67.7 ± 11.7	N/A	73/74	77/70	N/A	2004-2014	6-24	Radiofrequency, cryoablation	LAA, RAA	MVS, TVS	147	147	N/A	Paroxysmal, persistent, or permanent AF
31	Niv Ad et al, 2017	Randomized controlled clinical trial	United States	186	70.7 ± 8.3	70.3 ± 9.5	N/A	72/21	71/22	N/A	2005-2015	6-24	Cut and sew, radiofrequency, cyroablation	PVI, LAA, RAA	AVS,CABG, MVS	93	93	N/A	Paroxysmal AF
32	Yang et al, 2017	Non-randomized retrospective study, Cohort	China	129	47.3 ± 9.9	52.5 ± 10.3	N/A	32/62	10/25	N/A	2006-2015	6-72	Radiofrequency, cryoablation	PVI, LAA, RAA	MVS, TVS	94	35	N/A	Long-standing persistent AF
33	Takai et al, 2017	Non-randomized retrospective study, Cohort	Japan	3342	70 ± 9.5	70 ± 11	72 ± 9.1	905/434	160/106	1140/657	2012	6-14	Cut and sew, radiofrequency, cyroablation	PVI, LAA, RAA	CABG	1339	266	1797	Paroxysmal AF and persistent AF
34	Bogachev et al, 2018	Prospective, Randomised	Russia	140	51.4 ± 69.8	50.9 ± 69.2	N/A	25/45	27/43	N/A	2014-2017	24	Radiofrequency, cryoablation	PVI, LAA, RAA	MVS	70	70	N/A	Paroxysmal AF
35	Yoshikawa et al, 2006	Cohort Retrospective	Japan	100	58.7 ± 9.1	60.3 ± 10.0	N/A	20/9	36/35	N/A	1997 - 2004	N/A	Radiofrequency and cryoablation	LAA and BAA with modified Cox-Maze III	AVR,AVr,MVR,MVr and CABG	29	71	N/A	Persistent or Permanent AF
36	Kalybekova et al, 2021	Prospective Randomized Trial	Russia	116	65 [61; 67.75]	62 [58; 66]	N/A	48/10	49/9	N/A	2016 - 2020	1, 2, 12, 24, 60	cut and sew	Cox-Maze IV, LAA, RAA, PVI	CABG	58	58	N/A	Persistent AF
37	DeRose, Jr et al, 2019	Multicenter Randomized Contriolled Trial	Canada	126	N/A	N/A	N/A	N/A	N/A	N/A	2010-2013	12	Radiofrequency and cryoablation	LAA, RAA, PVI	MVS, CABG, AVS, TVS	64	N/A	62	Persistent AF
38	De Lima et al, 2004	Randomized Controlled Trial	Brazil	20	50.1 ± 15.3	N/A	54.1 ± 9.4	3/7	N/A	3/7	1999-2001	2, 6, 12, 18, 24	Electrocoagulation, cut and sew	Cox-Maze III, PVI	MVS	10	N/A	10	Permanent AF
39	Wang et al, 2014	Prospective Randomized Trial	China	210	53.1 ± 9.3	52.3 ± 10.3	N/A	23/47	28/42	N/A	2008-2011	3, 6, 12	Radiofrequency ablation	LAA, BAA	AVR, TVP, CABG	70	70	N/A	Chronic atrial fibrilation

Table S3. Postoperative Outcomes

No	Author, year	Non sinus rhytm (SR)			Pacemaker									Reoperation			Bleeding			Cerebrovascular Accidents			Renal failure			Overall-mortality		
					Temporary			Definitive/Permanent			Total																	
		BAA	LAA	PVI	BAA	LAA	PVI	BAA	LAA	PVI	BAA	LAA	PVI	BAA	LAA	PVI	BAA	LAA	PVI	BAA	LAA	PVI	BAA	LAA	PVI	BAA	LAA	PVI
1	Hald et al, 2021	3,12 months: 27 (25.0)	N/A	3,12 months: 105 (31.0)	N/A	N/A	N/A	13 (12)	N/A	20 (6)	13 (12)	N/A	20 (6)	N/A	N/A	N/A	N/A	N/A	N/A	N/A	N/A	N/A	N/A	N/A	N/A	N/A	N/A	N/A
2	Albrecht et al, 2009	Discharged: 5 (25.0)<br>Beyond 1 year: 3 (15.0)	N/A	Discharged: 4(20.0)<br>Beyond 1 year: 2 (10.0)	N/A	N/A	N/A	N/A	N/A	N/A	N/A	N/A	N/A	2 (10.0)	N/A	0 (0.0)	1 (5.0)	N/A	0 (0.0)	1 (5.0)	N/A	2 (10.0)	N/A	N/A	N/A	3 (15.0)	N/A	1 (5.0)
3	Srivastava et al, 2008	Discharged: 23 (57.5)<br>3,6 months: 23 (57.5)	Discharged: 21 (52.5)<br>3,6 months: 22 (55.0)	Discharged: 20 (50.0)<br>3,6 months: 22 (55.0)	0 (0.0)	1 (2.5)	0 (0.0)	0 (0.0)	0 (0.0)	0 (0.0)	0 (0.0)	1 (2.5)	0 (0.0)	1 (2.5)	2 (5.0)	0 (0.0)	1 (2.5)	2 (5.0)	0 (0.0)	N/A	N/A	N/A	N/A	N/A	N/A	4 (10.0)	3 (7.5)	4 (10.0)
4	Stulak et al, 2014	Beyond 1 year: 103(20.0)	Beyond 1 year: 24 (21.9)	Beyond 1 year: 100 (37.2)	N/A	N/A	N/A	12 (2.3)	3 (2.7)	7 (2.6)	12 (2.3)	3 (2.7)	7 (2.6)	33 (6.4)	1 (1.0)	8 (3.0)	33 (6.4)	1 (1.0)	8 (3.0)	6 (1.0)	0 (0.0)	1 (1.0)	10 (1.9)	3 (2.7)	9 (3.3)	12 (2.3)	12 (10.9)	28 (10.4)
5	Onorati et al, 2011	In-hospital postoperative: 10 (31.0)<br>Discharged (n: 32): 5 (5.0)<br>3 months (n:32): 20 (19.0)<br>6 months (n:32): 8 (8.0)<br>2 months (n:31) :17 (17.0)<br>Beyond 1 year (18 months) (n: 29) :10 (16.0)<br>Beyond 1 year (24 months) (n: 26) : 8 (16.0)	In-hospital postoperative: 10 (31.0)<br>Discharged (n: 32): 5 (5.0)<br>3 months (n:32): 20 (62%)<br>6 months (n:32): 8 (25.0)<br>12 months (n:31) : 11 (35.0)<br>Beyond 1 year (18 months) (n: 29): 12 (41.0)<br>Beyond 1 year (24 months) (n: 26) : 10 (38.0)	N/A	N/A	N/A	N/A	N/A	N/A	N/A	N/A	N/A	N/A	N/A	N/A	N/A	N/A	N/A	N/A	N/A	N/A	N/A	N/A	N/A	N/A	5 (4.6)	5 (15.6)	N/A
6	Alexander et al, 2018	N/A	Discharged: 6 (24.0)<br>Beyond 1 year: 2 (8.0)	Discharged: 17(62.9)<br>Beyond 1 year :12 (44.4)	N/A	N/A	N/A	N/A	1(4.0)	N/A	N/A	1(4.0)	N/A	N/A	N/A	N/A	N/A	1 (4.0)	1 (3.7)	N/A	N/A	N/A	N/A	N/A	N/A	N/A	N/A	1 (3.7)
7	Gauzebroek et al, 2008	Beyond 1 year: 10 (40.0)	Beyond 1 year: 12 (41.0)	Beyond 1 year: 8 (66.7)	16 (61.5)	7 (25.0)	2 (17.0)	N/A	1 (3.6)	N/A	16 (61.5)	8 (28.5)	2 (17.0)	N/A	N/A	N/A	N/A	N/A	N/A	N/A	N/A	N/A	N/A	N/A	N/A	2 (7.7)	N/A	N/A
8	Ghadivel et al, 2008	In-hospital postoperative: 2 (8.0)<br>12 months : 10 (40.0)	In-hospital postoperative : 0 (0.0)<br>12 months :20 (30.8)	N/A	N/A	N/A	N/A	N/A	N/A	N/A	N/A	N/A	N/A	N/A	N/A	N/A	N/A	N/A	N/A	N/A	N/A	N/A	N/A	N/A	N/A	1 (4.0)	2 (3.0)	N/A
9	Gillinov et al, 2015	6,12 months: 16 (34.0)	N/A	6,12 months: 23 (38.9)	N/A	N/A	N/A	N/A	N/A	N/A	N/A	N/A	N/A	N/A	N/A	N/A	N/A	N/A	N/A	N/A	N/A	N/A	N/A	N/A	N/A	N/A	N/A	N/A
10	Yildirim et al, 2022	N/A	Discharged: 29 (34)<br>3 months: 23 (27)<br>12 months: 21 (24)	Discharged: 58(32)<br>3 months: 55 (30)<br>12 months: 55 (30)	N/A	N/A	N//A	N/A	6 (6.4)	9 (5.0)	N/A	N/A	N/A	N/A	N/A	N/A	N/A	N/A	N/A	N/A	N/A	N/A	N/A	N/A	N/A	N/A	in-hospital<br>2 (2.8)<br><br>1 year<br>6 (6.5)	in-hospital<br>6 (3.4)<br><br>1 year<br>10 (5.3)
11	Wynn et al, 2016	12 months: 24 (38.0)	N/A	12 months: 21 (34.4)	N/A	N/A	N/A	N/A	N/A	N/A	N/A	N/A	N/A	N/A	N/A	N/A	N/A	N/A	N/A	N/A	N/A	N/A	N/A	N/A	N/A	N/A	N/A	1 (1.6)
12	Wang et al, 2009	Discharged: 33 (22.0)<br>12 months: 38 (25.3)<br>Beyond 1 year: 34 (22.7)	Discharged: 36 (24.2)<br>12 months: 39 (26.1)<br>Beyond 1 year: 28 (18.8)	N/A	N/A	N/A	N/A	2 (1.3)	1 (0.7)	N/A	2 (1.3)	1 (0.7)	N/A	N/A	N/A	N/A	4(2.7)	5 (3.4)	N/A	N/A	N/A	N/A	N/A	N/A	N/A	7 (4.7)	2 (1.3)	N/A
13	Calo et al, 2006	Beyond 1 year: 6 (15.4)	Beyond 1 year: 16 (39.0)	N/A	N/A	N/A	N/A	N/A	N/A	N/A	N/A	N/A	N/A	N/A	N/A	N/A	N/A	N/A	N/A	N/A	N/A	N/A	N/A	N/A	N/A	N/A	N/A	N/A
14	Henn et al, 2015	Discharged: 4 (6.6)	Discharged: 10 (20.8)	N/A	N/A	N/A	N/A	N/A	N/A	N/A	N/A	N/A	N/A	N/A	N/A	N/A	N/A	N/A	N/A	N/A	N/A	N/A	N/A	N/A	N/A	N/A	N/A	N/A
15	Albage et al, 2011	Beyond 1 year: 8 (18.2)	Beyond 1 year: 20 (28.2)	N/A	N/A	N/A	N/A	5 (11.4)	6 (8.5)	N/A	5 (11.4)	6 (8.5)	N/A	N/A	N/A	N/A	4 (9.0)	2 (2.8)	N/A	1 (2.3)	2 (2.8)	N/A	N/A	N/A	N/A	0 (0.0)	4 (5.6)	N/A
16	McCarthy et al, 2010	12 months: 14 (15.4)<br>Beyond 1 year: 12 (13.2)	12 months: 26 (14.6)<br>Beyond 1 year : 34 (19.4)	12 months: 14 (28.0)<br>Beyond 1 yeer: 14 (28.0)	N/A	N/A	N/A	N/A	N/A	N/A	N/A	N/A	N/A	29 (31.9)	47 (26.9)	4 (8.0)	0 (0.0)	7 (4.0)	2 (4.0)	2 (2.2)	1 (0.5)	0 (0.0)	N/A	N/A	N/A	2 (2.2)	8 (4.6)	2 (4.0)
17	Guden et al, 2003	Beyond 1 year: 9 (18.8)	Beyond 1 year: 13 (22.8)	N/A	10 (20.8)	10 (17.5)	N/A	2 (4.1)	0 (0.0)	N/A	12 (25.0)	10 (17.5)	N/A	3 (6.2)	1 (1.7)	N/A	3 (6.2)	1 (1.7)	N/A	N/A	N/A	N/A	N/A	N/A	N/A	4 (8.3)	2 (3.5)	N/A
18	Pecha et al, 2014a	Discharged: 27 (20.6)	Discharged: 77 (16.7)	N/A	N/A	N/A	N/A	15 (11.5)	26 (5.6)	N/A	15 (11.5)	26 (5.6)	N/A	N/A	N/A	N/A	N/A	N/A	N/A	N/A	N/A	N/A	N/A	N/A	N/A	N/A	N/A	N/A
19	Gualis et al, 2016	Discharged: 2 (2.9)<br>6 months: 4 (6.0)<br>12 months: 7 (10.4)<br>Beyond 1 year: 13 (19.4)	Discharged: 5 (6.0)<br>6 months: 9 (10.8)<br>12 months: 2 (26.5)<br>Beyond 1 year: 34 (41.0)	N/A	N/A	N/A	N/A	N/A	N/A	N/A	N/A	N/A	N/A	N/A	N/A	N/A	N/A	N/A	N/A	N/A	N/A	N/A	N/A	N/A	N/A	N/A	N/A	N/A
20	Deneke et al, 2009	Beyond 1 year: 22 (34.3)	Beyond 1 year: 18 (27.3)	N/A	N/A	N/A	N/A	N/A	N/A	N/A	N/A	N/A	N/A	N/A	N/A	N/A	N/A	N/A	N/A	N/A	N/A	N/A	N/A	N/A	N/A	N/A	N/A	N/A
21	Pecha et al, 2014b	Discharged : 23 (37.9)<br>3 months : 22 (26.1)<br>6 months: 12 (22.7)<br>12 months: 16 (25.6)	Discharged : 32 (48.9)<br>3 months : 29 (46.8)<br>6 months: 14 (43.7)<br>12 months: 28 (44.2)	N/A	N/A	N/A	N/A	7 (10.6)	4 (6.1)	N/A	7 (10.6)	4 (6.1)	N/A	N/A	N/A	N/A	N/A	N/A	N/A	N/A	N/A	N/A	N/A	N/A	N/A	4 (6.1)	3 (4.5)	N/A
22	Soni et al, 2013	3 months: 18 (19.8)<br>6 months: 22 (24.2)<br>12 months: 17 (18.7)	3 months: 77 (36.0)<br>6 months: 80 (37.4)<br>12 months: 80 (37.4)	N/A	N/A	N/A	N/A	15 (16.5)	16 (7.5)	N/A	15 (16.5)	16 (7.5)	N/A	N/A	N/A	N/A	N/A	N/A	N/A	N/A	N/A	N/A	N/A	N/A	N/A	3 (3.3)	7 (3.3)	N/A
23	Kim et al, 2011	3 months: 18 (9.0)<br>6 months: 16 (8.0)<br>12 months: 13 (6.5)<br>Beyond 1 year: 8 (4.0)	3 months: 7 (8.2)<br>6 months: 6 (7.1)<br>12 months: 9 (10.6)<br>Beyond 1 year: 2 (2.4)	N/A	N/A	N/A	N/A	3 (1.5)	0 (0.0)	N/A	3 (1.5)	0 (0.0)	N/A	2 (1.0)	1 (1.2)	N/A	12 (6.0)	0 (0.0)	N/A	3 (1.5)	14 (16.5)	N/A	N/A	N/A	N/A	12 (6.0)	2 (2.4)	N/A
24	Breda et al, 2011	Discharged: 3 (20.0)<br>6 months: 4 (26.6)	Discharged: 6 (20.0)<br>6 months: 8 (53.3)	N/A	1 (6.7)	N/A	N/A	N/A	N/A	N/A	N/A	N/A	N/A	N/A	N/A	N/A	N/A	N/A	N/A	N/A	N/A	N/A	N/A	N/A	N/A	0 (0.0)	0 (0.0)	N/A
25	Deneke et al, 2007	6 months: 28 (26.4)	6 months: 20 (17.2)	N/A	N/A	N/A	N/A	N/A	N/A	N/A	N/A	N/A	N/A	N/A	N/A	N/A	N/A	N/A	N/A	N/A	N/A	N/A	N/A	N/A	N/A	1 (0.9)	1(0.9)	N/A
26	Ryan et al, 2004	Beyond 1 year: 7 (19.4)	Beyond 1 year: 2 (28.6)	Beyond 1 year: 1 (6.7)	N/A	N/A	N/A	N/A	N/A	N/A	N/A	N/A	N/A	N/A	N/A	N/A	N/A	N/A	N/A	N/A	N/A	N/A	N/A	N/A	N/A	N/A	N/A	N/A
27	Takami et al, 2019	Discharged: 12 (40.0)<br>6 months: 5 (16.7)	Discharged: 7 (35.0)<br>6 months: 4 (20.0)	N/A	N/A	N/A	N/A	2 (6.7)	1 (5.0)	N/A	2 (6.7)	1 (5.0)	N/A	N/A	N/A	N/A	N/A	N/A	N/A	N/A	N/A	N/A	N/A	N/A	N/A	N/A	N/A	N/A
28	Takasaki et al, 2012	Beyond 1 year: 9 (25.7)	N/A	Beyond 1 year: 14 (35.0)	N/A	N/A	N/A	1 (2.9)	N/A	1 (2.5)	1 (2.9)	N/A	1 (2.5)	N/A	N/A	N/A	N/A	N/A	N/A	N/A	N/A	N/A	N/A	N/A	N/A	N/A	N/A	N/A
29	Charitos et al, 2015	N/A	N/A	N/A	N/A	N/A	N/A	N/A	N/A	N/A	N/A	N/A	N/A	N/A	N/A	N/A	0 (0.0)	0 (0.0)	N/A	1 (3.4)	0 (0.0)	N/A	N/A	N/A	N/A	N/A	N/A	N/A
30	Churyla et al, 2017	Discharged: 15 (10.2)<br>Beyond 1 year: 50 (34.0)	Discharged: 4 (2.8)<br>Beyond 1 year: 43 (29.3)	N/A	N/A	N/A	N/A	19 (13.0)	10 (7.0)	N/A	19 (13.0)	10 (7.0)	N/A	N/A	N/A	N/A	N/A	N/A	N/A	N/A	N/A	N/A	N/A	N/A	N/A	4 (2.7)	4 (2.7)	N/A
31	Niv Ad et al, 2017	6 months: 23 (24.7)<br>12 months: 16 (17.2)<br>Beyond 1 year: 13 (14.0)	6 months: 19 (20.4)<br>12 months: 14 (15.1)<br>Beyond 1 year: 23 (24.7)	N/A	N/A	N/A	N/A	N/A	7 (7.5)	N/A	N/A	7 (7.5)	N/A	N/A	1 (1.1)	N/A	N/A	1 (1.1)	N/A	6 (6.5)	9 (9.7)	N/A	N/A	1 (1.1)	N/A	N/A	1 (1.1)	N/A
32	Yang et al, 2017	Discharged: 16 (17.0)<br>6 months: 13 (13.8)<br>12 months: 14 (14.9)<br>Beyond 1 year (2 years): 14 (14.9<br>Beyond 1 year (3 years): 13 (13.8<br>Beyond 1 year (4 years): 20 (21.2<br>Beyond 1 year (6 years) :16 (17.0	Discharged: 7 (20.0)<br>6 months: 5 (14.3)<br>12 months: 7 (20.0)<br>Beyond 1 year (2 years): 5 (14.3)<br>Beyond 1 year (3 years): 6 (17.1)<br>Beyond 1 year (4 years): 6 (17.1)<br>Beyond 1 year (6 years) :7 (20.0)	N/A	N/A	N/A	N/A	N/A	N/A	N/A	N/A	N/A	N/A	N/A	N/A	N/A	4 (4.3)	2 (5.7)	N/A	7 (7.4)	1 (2.9)	N/A	6 (6.4)	2 (5.7)	N/A	4 (4.3)	1 (2.9)	N/A
33	Takai et al, 2017	Beyond 1 year: 416 (31.1)	Beyond 1 year: 87 (32,7)	Beyond 1 year: 601 (33.4)	N/A	N/A	N/A	N/A	N/A	N/A	N/A	N/A	N/A	27 (2.0)	7 (2.6)	16 (0.9)	37 (2.8)	3 (1.1)	59 (3.3)	68 (5.1)	16 (6.0)	108 (6.0)	339 (25.3)	58 (21.8)	463 (25.8)	19 (1.4)	5 (1.9)	36 (2.0)
34	Bogachev et al, 2018	Discharged: 4 (5.7)<br>3 months: 3 (4.3)<br>6 months: 5 (7.1)<br>12 months: 7 (10.0)<br>yond 1 year (18 months): 10 (14<br>Beyond 1 year (2 years): 14 (20.0	Discharged: 2 (2.9)<br>3 months: 2 (2.9)<br>6 months: 3 (4.3)<br>12 months: 3 (4.3)<br>eyond 1 year (18 months): 5 (7.1<br>Beyond 1 year (2 years): 5 (7.1)	N/A	N/A	N/A	N/A	8 (11.4)	4 (5.7)	N/A	8 (11.4)	4 (5.7)	N/A	N/A	N/A	N/A	2 (2.9)	3 (4.3)	N/A	3 (4.3)	1 (1.5)	N/A	N/A	N/A	N/A	2 (2.9)	1 (1.5)	N/A
35	Yoshikawa et al, 2006	Discharged: 6 (21.4)	Discharged: 20 (28.3)	N/A	N/A	N/A	N/A	N/A	N/A	N/A	N/A	N/A	N/A	N/A	N/A	N/A	N/A	N/A	N/A	N/A	N/A	N/A	N/A	N/A	N/A	N/A	N/A	N/A
36	Kalybekova et al, 2021	yond 1 year (24 months): 22 (38	yond 1 year (24 months): 37 (64	N/A	5 (8.6)	2 (3.5)	N/A	1 (1.7)	1 (1.7)	N/A	6 (10.3)	3 (5.2)	N/A	N/A	N/A	N/A	N/A	N/A	N/A	N/A	N/A	N/A	N/A	N/A	N/A	N/A	N/A	N/A
37	DeRose, Jr et al, 2019	N/A	N/A	N/A	N/A	N/A	N/A	16 (45.7)	N/A	10 (28.6)	16 (45.7)	N/A	10 (28.6)	N/A	N/A	N/A	N/A	N/A	N/A	N/A	N/A	N/A	N/A	N/A	N/A	N/A	N/A	N/A
38	De Lima et al, 2004	Beyond 1 year (18 months): 2 (20.0)	N/A	Beyond 1 year (18 months): 1 (10.0)	N/A	N/A	N/A	0 (0.0)	N/A	0 (0.0)	0 (0.0)	N/A	0 (0.0)	N/A	N/A	N/A	N/A	N/A	N/A	N/A	N/A	N/A	N/A	N/A	N/A	1 (10)	N/A	0 (0.0)
39	Wang et al, 2014	6 months: 43 (61.4)<br>12 months: 40 (57.1)	6 months: 36 (51.4)<br>12 months: 26 (37.1)	N/A	N/A	N/A	N/A	3 (4.3)	2 (2.9)	N/A	3 (4.3)	2 (2.9)	N/A	N/A	N/A	N/A	N/A	N/A	N/A	N/A	N/A	N/A	N/A	N/A	N/A	1 (1.4)	0 (0.0)	N/A

Table S4. Operative Procedure Time

No	Author, year	Xc time (Mean+/-SD or Range)			CPB/ECC time (Mean+/-SD or Range)			Ablation time (Mean+/-SD or Range)		
		BAA	LAA	PVI	BAA	LAA	PVI	BAA	LAA	PVI
1	Hald et al, 2021	N/A	N/A	N/A	N/A	N/A	N/A	N/A	N/A	N/A
2	Albrecht et al, 2009	N/A	N/A	N/A	122.95 ± 21.0	N/A	99.85 ± 23.8	N/A	N/A	N/A
3	Srivastava et al, 2008	N/A	N/A	N/A	N/A	N/A	N/A	17.75 ± 4.32	7.1 ± 1.57	5 ± 1.72
4	Stulak et al, 2014	59 ± 5	81 ± 6	65 ± 6	103 ± 7	109 ± 7	90 ± 8	N/A	N/A	N/A
5	Onorati et al, 2011	74 ± 6	73 ± 7	N/A	112 (103–133)	110 (102–113)	N/A	N/A	N/A	N/A
6	Alexander et al, 2018	N/A	78.7 ± 16.5	63.2 ± 12.3	N/A	99.2 ± 13.4	79.7 ± 10.1	N/A	23.8 ± 4.1	7.1 ± 2.3
7	Gauzebroek et al, 2008	94 ± 41	83 ± 34	68 ± 28	147 ± 49	126 ± 42	102 ± 28	N/A	N/A	N/A
8	Ghadivel et al, 2008	N/A	N/A	N/A	141 ± 40	120 ± 37	N/A	13 ± 5.1	10 ± 3.5	N/A
9	Gillinov et al, 2015	N/A	N/A	N/A	N/A	N/A	N/A	N/A	N/A	N/A
10	Yildirim et al, 2022	N/A	89 ± 9	71 ± 8	N/A	N/A	N/A	N/A	N/A	N/A
11	Wynn et al, 2016	N/A	N/A	N/A	209 ± 52	N/A	172 ± 44	72.5 ± 18	N/A	41.7 ± 14.6
12	Wang et al, 2009	80 ± 30	80 ± 33	N/A	128 ± 40	114 ± 41	N/A	29 ± 2	21 ± 3	N/A
13	Calo et al, 2006	N/A	N/A	N/A	228 ± 32	164 ± 25	N/A	41 ± 14	31 ± 1	N/A
14	Henn et al, 2015	N/A	N/A	N/A	N/A	N/A	N/A	N/A	N/A	N/A
15	Albage et al, 2011	N/A	N/A	N/A	N/A	N/A	N/A	N/A	N/A	N/A
16	McCarthy et al, 2010	N/A	N/A	N/A	N/A	N/A	N/A	N/A	N/A	N/A
17	Guden et al, 2003	N/A	N/A	N/A	N/A	N/A	N/A	N/A	N/A	N/A
18	Pecha et al, 2014a	N/A	N/A	N/A	N/A	N/A	N/A	N/A	N/A	N/A
19	Gualis et al, 2016	N/A	N/A	N/A	N/A	N/A	N/A	N/A	N/A	N/A
20	Deneke et al, 2009	88 ± 25	90 ± 19	N/A	149 ± 36	135 ± 28	N/A	N/A	N/A	N/A
21	Pecha et al, 2014b	N/A	N/A	N/A	N/A	N/A	N/A	N/A	N/A	N/A
22	Soni et al, 2013	98 ± 41	86 ± 41	N/A	152 ± 34	136 ± 54	N/A	N/A	N/A	N/A
23	Kim et al, 2011	111.9 ± 36.9	103.7 ± 28.5	N/A	171.3 ± 57.4	165.6 ± 46.3	N/A	N/A	N/A	N/A
24	Breda et al, 2011	72.33 ± 22.95	34.04 ± 11.17	N/A	100 ± 23.52	50.65 ± 15.17	N/A	7 ± 0.63	4 ± 0.78	N/A
25	Deneke et al, 2007	101 ± 21	99 ± 22	N/A	N/A	N/A	N/A	N/A	N/A	N/A
26	Ryan et al, 2004	N/A	N/A	N/A	N/A	N/A	N/A	N/A	N/A	N/A
27	Takami et al, 2019	152.2 ± 28.2	134.9 ± 15.8	N/A	248.2 ± 59.0	200.1 ± 25.7	N/A	N/A	N/A	N/A
28	Takasaki et al, 2012	N/A	N/A	N/A	143 ± 37	N/A	138 ± 41	N/A	N/A	N/A
29	Charitos et al, 2015	N/A	N/A	N/A	N/A	N/A	N/A	N/A	N/A	N/A
30	Churyla et al, 2017	93 (77–120)	99 (76–123)	N/A	132 (110–160)	134 (110–160)	N/A	N/A	N/A	N/A
31	Niv Ad et al, 2017	N/A	N/A	N/A	N/A	N/A	N/A	N/A	N/A	N/A

Table S4. Operative Procedure Time

No	Author, year	Xc time (Mean+/-SD or Range)			CPB/ECC time (Mean+/-SD or Range)			Ablation time (Mean+/-SD or Range)		
		BAA	LAA	PVI	BAA	LAA	PVI	BAA	LAA	PVI
32	Yang et al, 2017	92.3 ± 29.0	88.2 ± 24.5	N/A	151.8 ± 45.0	145.4 ± 50.0	N/A	N/A	N/A	N/A
33	Takai et al, 2017	127 ± 50	127 ± 52	109 ± 47	190 ± 67	187 ± 80	171 ± 71	N/A	N/A	N/A
34	Bogachev et al, 2018	107.9 ± 25.6	131.8 ± 30.9	N/A	N/A	N/A	N/A	N/A	N/A	N/A
35	Yoshikawa et al, 2006	131 ± 41.7	129 ± 34.2	N/A	187.5 ± 44.6	187.0 ± 48.6	N//A	N/A	N/A	N/A
36	Kalybekova et al, 2021	N/A	N/A	N/A	N/A	N/A	N/A	N/A	N/A	N/A
37	DeRose, Jr et al, 2019	N/A	N/A	N/A	N/A	N/A	N/A	N/A	N/A	N/A
38	De Lima et al, 2004	N/A	N/A	N/A	115.3 ± 25	N/A	97.8 ± 3	N/A	N/A	N/A
39	Wang et al, 2014	N/A	N/A	N/A	138.2 ± 46.0	101.0 ± 34.0	N/A	N/A	N/A	N/A

Table S5. Hospital Admissions and Stay

No	Author, year	Hospital stay			Hospital Redmissions		
		BAA	LAA	PVI	BAA	LAA	PVI
1	Hald et al, 2021	N/A	N/A	N/A	N/A	N/A	N/A
2	Albrecht et al, 2009	N/A	N/A	N/A	N/A	N/A	N/A
3	Srivastava et al, 2008	N/A	N/A	N/A	N/A	N/A	N/A
4	Stulak et al, 2014	N/A	N/A	N/A	N/A	N/A	N/A
5	Onorati et al, 2011	N/A	N/A	N/A	14 (12.8)	13 (40.6)	N/A
6	Alexander et al, 2018	N/A	N/A	N/A	N/A	N/A	N/A
7	Gauzebroek et al, 2008	13.2 ± 6.0	10.7 ± 3.2	11.1 ± 5.6	N/A	N/A	N/A
8	Ghadivel et al, 2008	N/A	N/A	N/A	10 (40.0)	25 (38.5)	N/A
9	Gillinov et al, 2015	N/A	N/A	N/A	N/A	N/A	N/A
10	Yildirim et al, 2022	N/A	N/A	N/A	N/A	N/A	N/A
11	Wynn et al, 2016	N/A	N/A	N/A	6(9.5)	N/A	5 (8.2)
12	Wang et al, 2009	N/A	N/A	N/A	N/A	N/A	N/A
13	Calo et al, 2006	N/A	N/A	N/A	N/A	N/A	N/A
14	Henn et al, 2015	N/A	N/A	N/A	N/A	N/A	N/A
15	Albage et al, 2011	N/A	N/A	N/A	N/A	N/A	N/A
16	McCarthy et al, 2010	N/A	N/A	N/A	N/A	N/A	N/A
17	Guden et al, 2003	N/A	N/A	N/A	N/A	N/A	N/A
18	Pecha et al, 2014a	N/A	N/A	N/A	N/A	N/A	N/A
19	Gualis et al, 2016	N/A	N/A	N/A	N/A	N/A	N/A
20	Deneke et al, 2009	N/A	N/A	N/A	N/A	N/A	N/A
21	Pecha et al, 2014b	N/A	N/A	N/A	N/A	N/A	N/A
22	Soni et al, 2013	11.2 ± 10.9	10.5 ± 9.1	N/A	N/A	N/A	N/A
23	Kim et al, 2011	N/A	N/A	N/A	1 (0.5)	0 (0.0)	N/A
24	Breda et al, 2011	N/A	N/A	N/A	N/A	N/A	N/A
25	Deneke et al, 2007	N/A	N/A	N/A	N/A	N/A	N/A
26	Ryan et al, 2004	N/A	N/A	N/A	N/A	N/A	N/A
27	Takami et al, 2019	N/A	N/A	N/A	N/A	N/A	N/A
28	Takasaki et al, 2012	N/A	N/A	N/A	N/A	N/A	N/A

Table S5. Hospital Admissions and Stay

No	Author, year	Hospital stay			Hospital Redmissions		
		BAA	LAA	PVI	BAA	LAA	PVI
29	Charitos et al, 2015	N/A	N/A	N/A	N/A	N/A	N/A
30	Churyla et al, 2017	7 (5–10)	7 (6–10)	N/A	N/A	N/A	N/A
31	Niv Ad et al, 2017	N/A	6 (4-8)	N/A	N/A	17 (18.3)	N/A
32	Yang et al, 2017	N/A	N/A	N/A	N/A	N/A	N/A
33	Takai et al, 2017	27 ± 27	29 ± 33	28 ± 29	N/A	N/A	N/A
34	Bogachev et al, 2018	N/A	N/A	N/A	N/A	N/A	N/A
35	Yoshikawa et al, 2006	N/A	N/A	N/A	N/A	N/A	N/A
36	Kalybekova et al, 2021	N/A	N/A	N/A	N/A	N/A	N/A
37	DeRose, Jr et al, 2019	N/A	N/A	N/A	N/A	N/A	N/A
38	De Lima et al, 2004	N/A	N/A	N/A	N/A	N/A	N/A
39	Wang et al, 2014	N/A	N/A	N/A	N/A	N/A	N/A

Figure S1. Overall postoperative outcomes

A

Non-Sinus Rhytm

Forest Plot of Non-Sinus Rhytm (Conventional Pairwise Meta-Analysis)

Study or Subgroup	BAA Events	BAA Total	LAA Events	LAA Total	Weight	Odds Ratio IV, Random, 95% CI
**2.1.1 In-hospital;postoperative**						
Ghadivel et al, 2008	2	25	0	65	0.2%	13.94 [0.65, 301.03]
Onorati et al, 2011	10	109	10	32	1.3%	0.22 [0.08, 0.60]
**Subtotal (95% CI)**		**134**		**97**	**1.5%**	**1.35 [0.02, 75.28]**
Total events	12		10			
Heterogeneity: Tau^2 = 7.21; Chi^2 = 6.31, df = 1 (P = 0.01); I^2 = 84%						
Test for overall effect: Z = 0.15 (P = 0.88)						
**2.1.2 Discharged**						
Bogachev et al, 2018	4	70	2	70	0.6%	2.06 [0.37, 11.63]
Breda et al, 2011	3	15	6	15	0.6%	0.38 [0.07, 1.92]
Churyla et al, 2017	15	147	44	147	2.1%	0.27 [0.14, 0.50]
Gualis et al, 2016	2	67	5	83	0.6%	0.48 [0.09, 2.56]
Henn et al, 2015	4	61	10	48	1.0%	0.27 [0.08, 0.91]
Onorati et al, 2011	5	32	5	32	0.9%	1.00 [0.26, 3.86]
Pecha et al, 2014a	27	131	77	463	2.5%	1.30 [0.80, 2.12]
Pecha et al, 2014b	23	66	32	66	1.9%	0.57 [0.28, 1.14]
Srivastava et al, 2008	23	40	21	40	1.5%	1.22 [0.51, 2.96]
Takami et al, 2019	12	30	7	20	1.0%	1.24 [0.38, 4.00]
Wang et al, 2009	33	150	36	149	2.3%	0.89 [0.52, 1.52]
Yang et al, 2017	16	94	7	35	1.3%	0.82 [0.31, 2.20]
Yoshikawa et al, 2006	6	29	20	71	1.2%	0.67 [0.24, 1.88]
**Subtotal (95% CI)**		**932**		**1239**	**17.6%**	**0.72 [0.51, 1.04]**
Total events	173		272			
Heterogeneity: Tau^2 = 0.18; Chi^2 = 23.05, df = 12 (P = 0.03); I^2 = 48%						
Test for overall effect: Z = 1.77 (P = 0.08)						
**2.1.3 3 months**						
Bogachev et al, 2018	3	70	2	70	0.5%	1.52 [0.25, 9.40]
Kim et al, 2011	18	199	7	85	1.5%	1.11 [0.44, 2.76]
Onorati et al, 2011	20	32	20	32	1.3%	1.00 [0.36, 2.75]
Pecha et al, 2014b	22	66	29	66	1.9%	0.64 [0.31, 1.29]
Soni et al, 2013	18	91	77	214	2.2%	0.44 [0.24, 0.79]
Srivastava et al, 2008	23	40	22	40	1.5%	1.11 [0.46, 2.68]
**Subtotal (95% CI)**		**498**		**507**	**8.9%**	**0.73 [0.51, 1.06]**
Total events	104		157			
Heterogeneity: Tau^2 = 0.03; Chi^2 = 5.67, df = 5 (P = 0.34); I^2 = 12%						
Test for overall effect: Z = 1.64 (P = 0.10)						
**2.1.4 6 months**						
Bogachev et al, 2018	5	70	3	70	0.7%	1.72 [0.39, 7.48]
Breda et al, 2011	4	15	8	15	0.7%	0.32 [0.07, 1.47]
Deneke et al, 2007	28	106	20	116	2.0%	1.72 [0.90, 3.29]
Gualis et al, 2016	4	67	9	83	1.0%	0.52 [0.15, 1.78]
Kim et al, 2011	16	199	6	85	1.3%	1.15 [0.43, 3.05]
Niv Ad et al, 2017	23	93	19	93	1.9%	1.28 [0.64, 2.55]
Onorati et al, 2011	8	32	8	32	1.1%	1.00 [0.32, 3.10]
Pecha et al, 2014b	12	66	14	66	1.6%	0.83 [0.35, 1.95]
Soni et al, 2013	22	91	80	214	2.3%	0.53 [0.31, 0.93]
Srivastava et al, 2008	23	40	22	40	1.5%	1.11 [0.46, 2.68]
Takami et al, 2019	5	30	4	20	0.8%	0.80 [0.19, 3.43]
Wang et al, 2014	43	70	36	70	2.0%	1.50 [0.77, 2.94]
Yang et al, 2017	13	94	5	35	1.1%	0.96 [0.32, 2.93]
**Subtotal (95% CI)**		**973**		**939**	**18.1%**	**1.00 [0.76, 1.30]**
Total events	206		234			
Heterogeneity: Tau^2 = 0.03; Chi^2 = 13.72, df = 12 (P = 0.32); I^2 = 13%						
Test for overall effect: Z = 0.03 (P = 0.97)						
**2.1.5 12 months**						
Bogachev et al, 2018	7	70	3	70	0.8%	2.48 [0.61, 10.02]
Ghadivel et al, 2008	10	25	20	65	1.4%	1.50 [0.58, 3.91]
Gualis et al, 2016	7	67	2	83	0.7%	4.72 [0.95, 23.56]
Kim et al, 2011	13	199	9	85	1.5%	0.59 [0.24, 1.44]
McCarthy et al, 2010	14	91	26	175	1.9%	1.04 [0.51, 2.11]
Niv Ad et al, 2017	16	93	14	93	1.7%	1.17 [0.54, 2.57]
Onorati et al, 2011	17	31	11	31	1.3%	2.21 [0.80, 6.13]
Pecha et al, 2014b	16	66	28	66	1.8%	0.43 [0.21, 0.91]
Soni et al, 2013	17	91	80	214	2.2%	0.38 [0.21, 0.70]
Wang et al, 2009	38	150	39	149	2.4%	0.96 [0.57, 1.61]
Wang et al, 2014	40	70	26	70	2.0%	2.26 [1.15, 4.44]
Yang et al, 2017	14	94	7	35	1.3%	0.70 [0.26, 1.91]
**Subtotal (95% CI)**		**1047**		**1136**	**18.8%**	**1.05 [0.70, 1.56]**
Total events	209		265			
Heterogeneity: Tau^2 = 0.29; Chi^2 = 30.29, df = 11 (P = 0.001); I^2 = 64%						
Test for overall effect: Z = 0.23 (P = 0.82)						
**2.1.6 Beyond 1 year**						
Albage et al, 2011	8	44	20	71	1.4%	0.57 [0.22, 1.43]
Bogachev et al, 2018 18m	10	70	5	70	1.1%	2.17 [0.70, 6.70]
Bogachev et al, 2018 2y	14	70	5	70	1.2%	3.25 [1.10, 9.59]
Calo et al, 2006	6	39	16	41	1.2%	0.28 [0.10, 0.83]
Churyla et al, 2017	50	147	43	147	2.5%	1.25 [0.76, 2.04]
Deneke et al, 2009	22	64	18	66	1.8%	1.40 [0.66, 2.95]
Gauzebroek et al, 2008	10	25	12	28	1.2%	0.89 [0.30, 2.66]
Gualis et al, 2016	13	67	34	83	1.8%	0.35 [0.16, 0.73]
Guden et al, 2003	9	48	13	57	1.4%	0.78 [0.30, 2.03]
Kalybekova et al, 2021	22	58	37	58	1.8%	0.35 [0.16, 0.74]
Kim et al, 2011	8	199	2	85	0.7%	1.74 [0.36, 8.36]
McCarthy et al, 2010	12	91	34	175	1.9%	0.63 [0.31, 1.29]
Niv Ad et al, 2017	13	93	23	93	1.8%	0.49 [0.23, 1.05]
Onorati et al, 2011 18m	10	29	12	29	1.2%	0.75 [0.26, 2.16]
Onorati et al, 2011 24m	8	26	0	26	0.2%	24.35 [1.32, 448.50]
Ryan et al, 2004	7	36	2	7	0.5%	0.60 [0.10, 3.78]
Stulak et al, 2014	103	514	24	110	2.5%	0.90 [0.54, 1.48]
Takai et al, 2017	416	1339	87	266	3.1%	0.93 [0.70, 1.23]
Wang et al, 2009	34	150	28	149	2.3%	1.27 [0.72, 2.22]
Yang et al, 2017 2y	14	94	5	35	1.1%	1.05 [0.35, 3.17]
Yang et al, 2017 3y	30	94	6	35	1.3%	2.27 [0.85, 6.04]
Yang et al, 2017 4y	20	94	6	35	1.3%	1.31 [0.48, 3.58]
Yang et al, 2017 6y	16	94	7	35	1.3%	0.82 [0.31, 2.20]
Yang et al, 2017 8y	7	94	2	35	0.6%	1.33 [0.26, 6.72]
**Subtotal (95% CI)**		**3579**		**1806**	**35.1%**	**0.91 [0.72, 1.15]**
Total events	862		441			
Heterogeneity: Tau^2 = 0.14; Chi^2 = 43.65, df = 23 (P = 0.006); I^2 = 47%						
Test for overall effect: Z = 0.81 (P = 0.42)						
**Total (95% CI)**		**7163**		**5724**	**100.0%**	**0.88 [0.76, 1.02]**
Total events	1566		1379			
Heterogeneity: Tau^2 = 0.15; Chi^2 = 131.53, df = 69 (P < 0.00001); I^2 = 48%						
Test for overall effect: Z = 1.71 (P = 0.09)						
Test for subgroup differences: Chi^2 = 3.72, df = 5 (P = 0.59), I^2 = 0%						

Odds Ratio
IV, Random, 95% CI

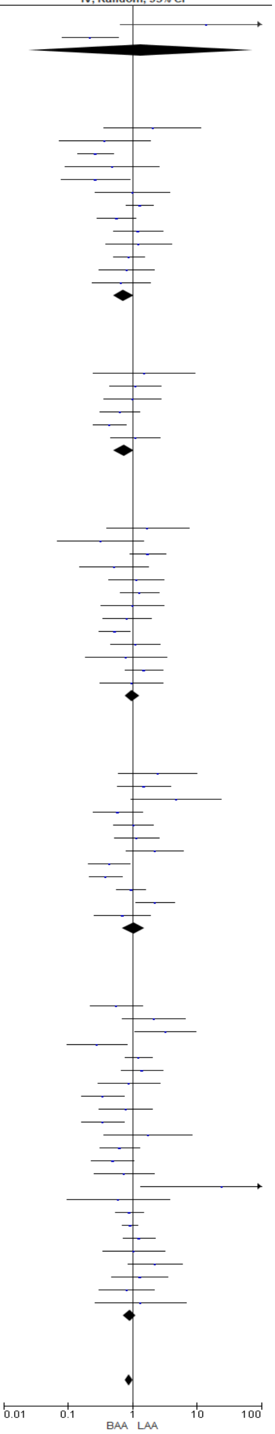

Study or Subgroup	BAA Events	BAA Total	PVI Events	PVI Total	Weight	Odds Ratio IV, Random, 95% CI
**1.1.2 Discharged**						
Albrecht et al, 2009	5	20	4	20	1.8%	1.33 [0.30, 5.93]
Srivastava et al, 2008	23	40	22	40	4.4%	1.11 [0.46, 2.68]
**Subtotal (95% CI)**		**60**		**60**	**6.1%**	**1.16 [0.54, 2.49]**
Total events	28		26			
Heterogeneity: Tau² = 0.00; Chi² = 0.04, df = 1 (P = 0.83); I² = 0%						
Test for overall effect: Z = 0.39 (P = 0.70)						
**1.1.3 3 months**						
Hald et al, 2021	27	109	105	337	9.7%	0.73 [0.44, 1.19]
Srivastava et al, 2008	23	40	27	40	4.2%	0.65 [0.26, 1.62]
**Subtotal (95% CI)**		**149**		**377**	**13.8%**	**0.71 [0.46, 1.09]**
Total events	50		132			
Heterogeneity: Tau² = 0.00; Chi² = 0.04, df = 1 (P = 0.83); I² = 0%						
Test for overall effect: Z = 1.55 (P = 0.12)						
**1.1.4 6 months**						
Gillinov et al, 2015	16	47	23	59	5.1%	0.81 [0.36, 1.80]
Srivastava et al, 2008	23	40	27	40	4.2%	0.65 [0.26, 1.62]
**Subtotal (95% CI)**		**87**		**99**	**9.3%**	**0.74 [0.40, 1.34]**
Total events	39		50			
Heterogeneity: Tau² = 0.00; Chi² = 0.12, df = 1 (P = 0.73); I² = 0%						
Test for overall effect: Z = 1.00 (P = 0.32)						
**1.1.5 12 months**						
Gillinov et al, 2015	16	47	23	59	5.1%	0.81 [0.36, 1.80]
Hald et al, 2021	27	109	105	337	9.7%	0.73 [0.44, 1.19]
McCarthy et al, 2010	14	91	14	50	4.7%	0.47 [0.20, 1.08]
Wynn et al, 2016	24	63	21	61	5.8%	1.17 [0.56, 2.44]
**Subtotal (95% CI)**		**310**		**507**	**25.3%**	**0.76 [0.55, 1.07]**
Total events	81		163			
Heterogeneity: Tau² = 0.00; Chi² = 2.68, df = 3 (P = 0.44); I² = 0%						
Test for overall effect: Z = 1.59 (P = 0.11)						
**1.1.6 Beyond 1 year**						
Albrecht et al, 2009	3	20	2	20	1.1%	1.59 [0.24, 10.70]
De Lima et al, 2004	2	10	1	10	0.6%	2.25 [0.17, 29.77]
Gauzebroek et al, 2008	10	25	8	12	1.9%	0.33 [0.08, 1.41]
McCarthy et al, 2010	12	91	14	50	4.5%	0.39 [0.16, 0.93]
Ryan et al, 2004	7	36	1	15	0.9%	3.38 [0.38, 30.20]
Stulak et al, 2014	103	514	100	269	13.9%	0.42 [0.30, 0.59]
Takai et al, 2017	411	1339	601	1797	19.1%	0.88 [0.76, 1.03]
Takasaki et al, 2012	9	35	14	40	3.6%	0.64 [0.24, 1.74]
**Subtotal (95% CI)**		**2070**		**2213**	**45.5%**	**0.65 [0.41, 1.03]**
Total events	557		741			
Heterogeneity: Tau² = 0.19; Chi² = 22.50, df = 7 (P = 0.002); I² = 69%						
Test for overall effect: Z = 1.84 (P = 0.07)						
**Total (95% CI)**		**2676**		**3256**	**100.0%**	**0.71 [0.58, 0.88]**
Total events	755		1112			
Heterogeneity: Tau² = 0.05; Chi² = 26.68, df = 17 (P = 0.06); I² = 36%						
Test for overall effect: Z = 3.19 (P = 0.001)						
Test for subgroup differences: Chi² = 1.74, df = 4 (P = 0.78), I² = 0%						

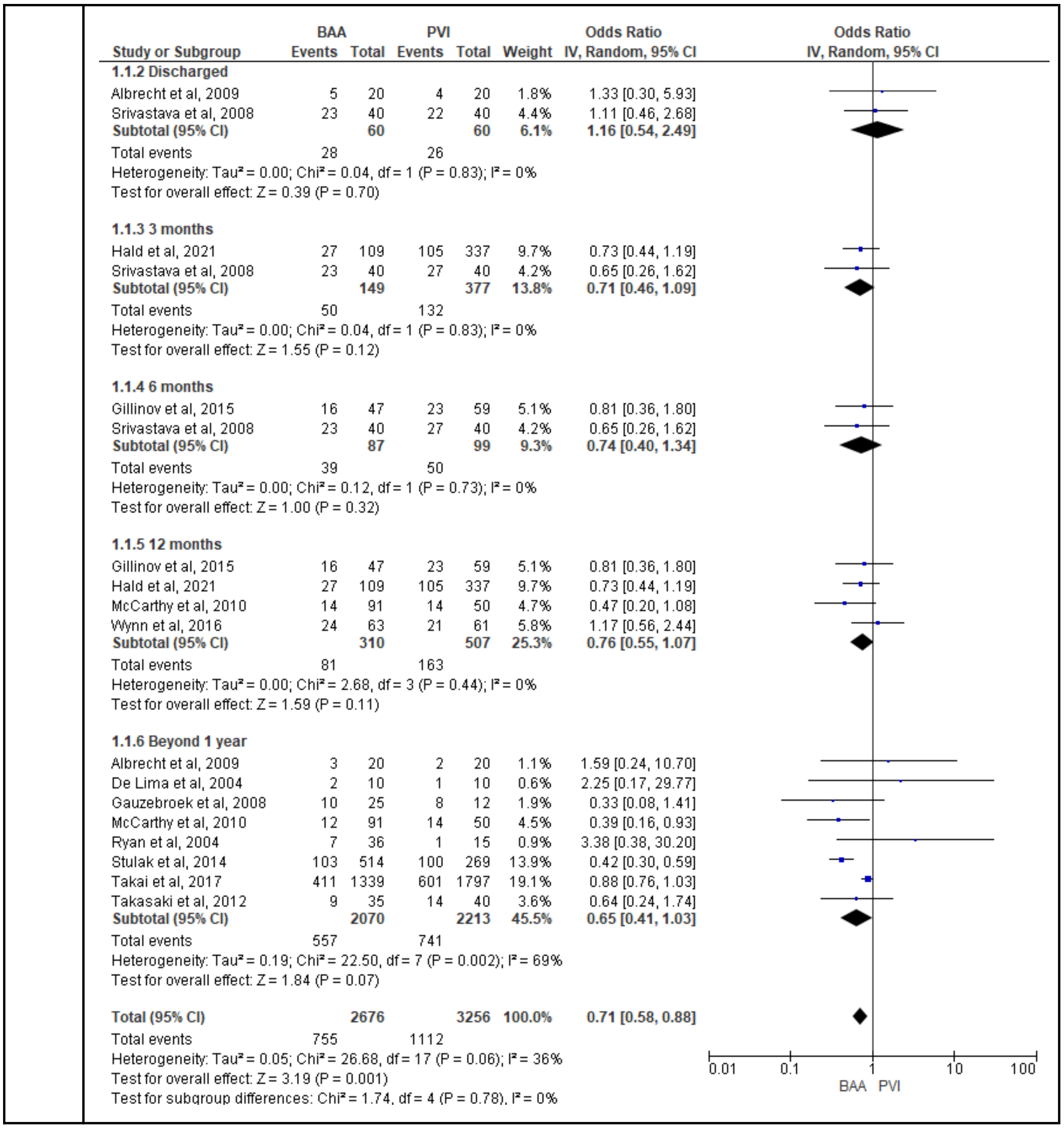

Study or Subgroup	LAA Events	LAA Total	PVI Events	PVI Total	Weight	Odds Ratio IV, Random, 95% CI
**3.1.2 Discharged**						
Alexander et al, 2018	6	25	17	27	4.4%	0.19 [0.06, 0.62]
Srivastava et al, 2008	21	40	20	40	6.8%	1.11 [0.46, 2.66]
Yildirim et al, 2022	29	86	58	182	10.9%	1.09 [0.63, 1.88]
**Subtotal (95% CI)**		**151**		**249**	**22.1%**	**0.69 [0.27, 1.74]**
Total events	56		95			
Heterogeneity: Tau² = 0.48; Chi² = 7.24, df = 2 (P = 0.03); I² = 72%						
Test for overall effect: Z = 0.79 (P = 0.43)						
**3.1.3 3 months**						
Srivastava et al, 2008	23	40	22	40	6.7%	1.11 [0.46, 2.68]
Yildirim et al, 2022	23	86	55	182	10.5%	0.84 [0.48, 1.49]
**Subtotal (95% CI)**		**126**		**222**	**17.2%**	**0.91 [0.57, 1.48]**
Total events	46		77			
Heterogeneity: Tau² = 0.00; Chi² = 0.26, df = 1 (P = 0.61); I² = 0%						
Test for overall effect: Z = 0.37 (P = 0.71)						
**3.1.4 6 months**						
Srivastava et al, 2008	23	40	22	40	0.0%	1.11 [0.46, 2.68]
**Subtotal (95% CI)**		**0**		**0**		**Not estimable**
Total events	0		0			
Heterogeneity: Not applicable						
Test for overall effect: Not applicable						
**3.1.5 12 months**						
McCarthy et al, 2010	26	175	14	50	8.2%	0.45 [0.21, 0.94]
Yildirim et al, 2022	21	86	55	182	10.3%	0.75 [0.42, 1.34]
**Subtotal (95% CI)**		**261**		**232**	**18.5%**	**0.61 [0.38, 0.99]**
Total events	47		69			
Heterogeneity: Tau² = 0.01; Chi² = 1.11, df = 1 (P = 0.29); I² = 10%						
Test for overall effect: Z = 1.98 (P = 0.05)						
**3.1.6 Beyond 1 year**						
Alexander et al, 2018	2	25	12	27	2.7%	0.11 [0.02, 0.56]
Gauzebroek et al, 2008	12	28	8	12	3.4%	0.38 [0.09, 1.54]
McCarthy et al, 2010	34	175	14	50	8.5%	0.62 [0.30, 1.28]
Ryan et al, 2004	2	7	1	15	1.2%	5.60 [0.41, 76.05]
Stulak et al, 2014	24	110	100	269	11.3%	0.47 [0.28, 0.79]
Takai et al, 2017	87	266	601	1797	15.2%	0.97 [0.74, 1.27]
**Subtotal (95% CI)**		**611**		**2170**	**42.3%**	**0.59 [0.33, 1.04]**
Total events	161		736			
Heterogeneity: Tau² = 0.26; Chi² = 15.22, df = 5 (P = 0.009); I² = 67%						
Test for overall effect: Z = 1.84 (P = 0.07)						
**Total (95% CI)**		**1149**		**2873**	**100.0%**	**0.69 [0.52, 0.93]**
Total events	310		977			
Heterogeneity: Tau² = 0.13; Chi² = 25.52, df = 12 (P = 0.01); I² = 53%						
Test for overall effect: Z = 2.48 (P = 0.01)						
Test for subgroup differences: Chi² = 1.84, df = 3 (P = 0.61), I² = 0%						

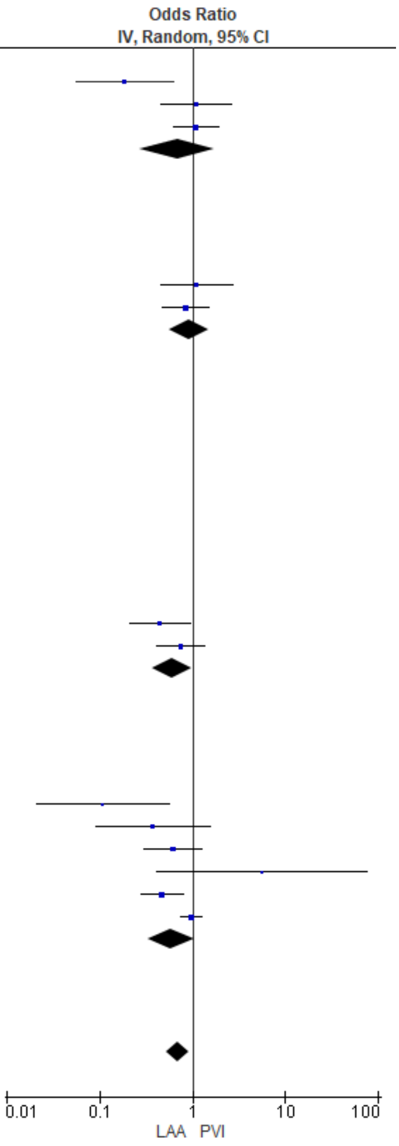

**B**	**Pacemaker Implantation**
	**Forest Plot of Pacemaker Implantation (Both temporary and permanent) (Conventional Pairwise Meta-Analysis)**

Study or Subgroup	BAA Events	BAA Total	Control Events	Control Total	Weight	Odds Ratio IV, Random, 95% CI
**1.1.1 BAA vs LAA**						
Albage et al, 2011	5	44	6	71	4.0%	1.39 [0.40, 4.85]
Bogachev et al, 2018	8	70	4	70	4.0%	2.13 [0.61, 7.43]
Churyla et al, 2017	19	147	10	147	9.7%	2.03 [0.91, 4.54]
Gauzebroek et al, 2008	16	25	8	28	4.7%	4.44 [1.40, 14.14]
Guden et al, 2003	12	48	10	57	7.0%	1.57 [0.61, 4.03]
Kalybekova et al, 2021	6	58	3	58	3.0%	2.12 [0.50, 8.90]
Kim et al, 2011	3	199	0	85	0.7%	3.05 [0.16, 59.61]
Pecha et al, 2014 (A)	15	131	26	463	14.0%	2.17 [1.11, 4.24]
Pecha et al, 2014 (B)	7	66	4	66	3.8%	1.84 [0.51, 6.61]
Soni et al, 2013	15	91	16	214	11.1%	2.44 [1.15, 5.18]
Srivastava et al, 2008	0	40	1	40	0.6%	0.33 [0.01, 8.22]
Stulak et al, 2014	12	514	3	110	3.8%	0.85 [0.24, 3.07]
Takami et al, 2019	2	30	1	20	1.0%	1.36 [0.11, 16.05]
Wang et al, 2009	2	150	1	149	1.1%	2.00 [0.18, 22.30]
Wang et al, 2014	3	70	2	70	1.9%	1.52 [0.25, 9.40]
**Subtotal (95% CI)**		**1683**		**1648**	**70.4%**	**1.98 [1.47, 2.67]**
Total events	125		95			
Heterogeneity: Tau² = 0.00; Chi² = 5.94, df = 14 (P = 0.97); I² = 0%						
Test for overall effect: Z = 4.49 (P < 0.00001)						
**1.1.2 BAA vs PVI**						
De Lima et al, 2004	0	10	0	10		Not estimable
DeRose, Jr et al, 2019	16	64	10	62	8.0%	1.73 [0.72, 4.19]
Gauzebroek et al, 2008	16	25	2	12	2.1%	8.89 [1.59, 49.83]
Hald et al, 2021	13	109	20	337	11.6%	2.15 [1.03, 4.47]
Srivastava et al, 2008	0	40	0	40		Not estimable
Stulak et al, 2014	12	514	7	269	7.0%	0.89 [0.35, 2.30]
Takasaki et al, 2012	1	35	1	40	0.8%	1.15 [0.07, 19.05]
**Subtotal (95% CI)**		**797**		**770**	**29.6%**	**1.83 [1.01, 3.31]**
Total events	58		40			
Heterogeneity: Tau² = 0.13; Chi² = 5.73, df = 4 (P = 0.22); I² = 30%						
Test for overall effect: Z = 1.99 (P = 0.05)						
**Total (95% CI)**		**2480**		**2418**	**100.0%**	**1.92 [1.50, 2.47]**
Total events	183		135			
Heterogeneity: Tau² = 0.00; Chi² = 11.80, df = 19 (P = 0.89); I² = 0%						
Test for overall effect: Z = 5.12 (P < 0.00001)						
Test for subgroup differences: Chi² = 0.05, df = 1 (P = 0.82), I² = 0%						

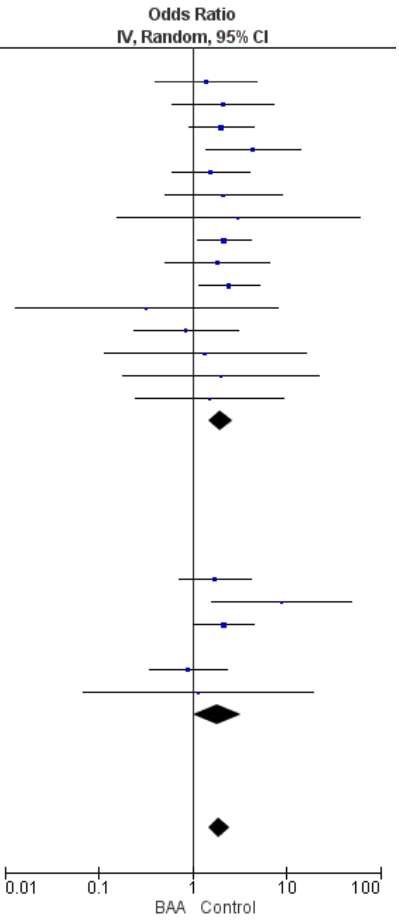

Study or Subgroup	LAA Events	LAA Total	PVI Events	PVI Total	Weight	Odds Ratio IV, Random, 95% CI
Gauzebroek et al, 2008	8	28	2	12	34.9%	2.00 [0.36, 11.23]
Srivastava et al, 2008	1	40	0	40	9.9%	3.08 [0.12, 77.80]
Stulak et al, 2014	3	110	7	269	55.2%	1.05 [0.27, 4.13]
**Total (95% CI)**		**178**		**321**	**100.0%**	**1.46 [0.53, 4.05]**
Total events	12		9			
Heterogeneity: Tau² = 0.00; Chi² = 0.56, df = 2 (P = 0.76); I² = 0%						
Test for overall effect: Z = 0.73 (P = 0.46)						

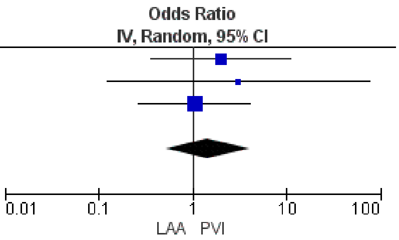

**C**	**Reoperation**
	**Forest Plot of Reoperation(Conventional Pairwise Meta-Analysis)**

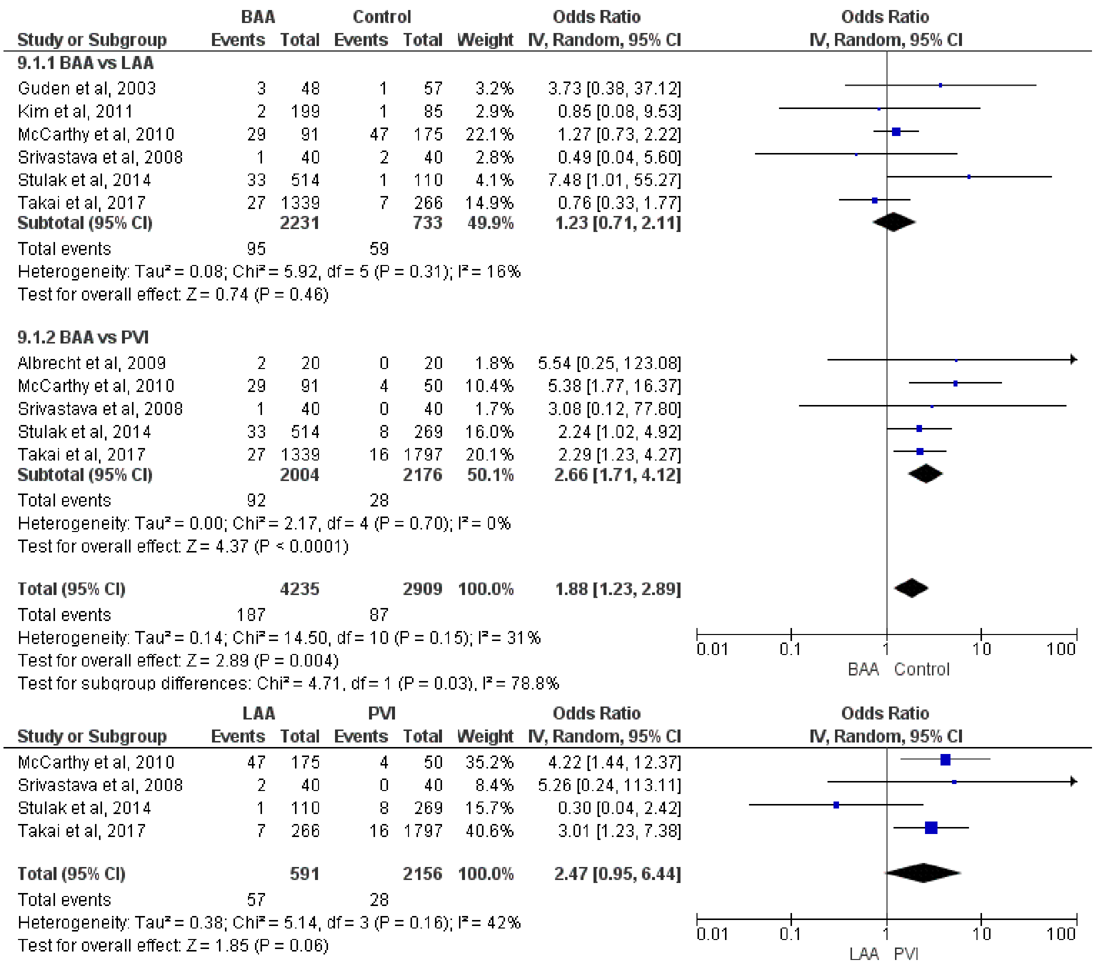

D	**Incidence of Bleeding**
	**Forest Plot of Bleeding(Conventional Pairwise Meta-Analysis)**

Study or Subgroup	BAA Events	BAA Total	Control Events	Control Total	Weight	Odds Ratio IV, Random, 95% CI
**11.1.1 BAA vs LAA**						
Albage et al, 2011	4	44	2	71	6.3%	3.45 [0.60, 19.69]
Bogachev et al, 2018	2	70	3	70	5.9%	0.66 [0.11, 4.06]
Charitos et al, 2015	0	29	0	22		Not estimable
Guden et al, 2003	3	48	1	57	4.1%	3.73 [0.38, 37.12]
Kim et al, 2011	12	199	0	85	2.8%	11.40 [0.67, 194.78]
McCarthy et al, 2010	0	91	7	175	2.8%	0.12 [0.01, 2.17]
Srivastava et al, 2008	1	40	2	40	3.7%	0.49 [0.04, 5.60]
Stulak et al, 2014	33	514	1	110	5.1%	7.48 [1.01, 55.27]
Takai et al, 2017	37	1339	3	266	10.5%	2.49 [0.76, 8.14]
Wang et al, 2009	4	150	5	149	9.1%	0.79 [0.21, 3.00]
Yang et al, 2017	4	94	2	35	6.3%	0.73 [0.13, 4.19]
**Subtotal (95% CI)**		**2618**		**1080**	**56.7%**	**1.55 [0.77, 3.13]**
Total events	100		26			
Heterogeneity: Tau^2 = 0.36; Chi^2 = 12.66, df = 9 (P = 0.18); I^2 = 29%						
Test for overall effect: Z = 1.23 (P = 0.22)						
**11.1.2 BAA vs PVI**						
Albrecht et al, 2009	1	20	0	20	2.2%	3.15 [0.12, 82.16]
McCarthy et al, 2010	0	91	2	50	2.5%	0.11 [0.00, 2.25]
Srivastava et al, 2008	1	40	0	40	2.3%	3.08 [0.12, 77.80]
Stulak et al, 2014	33	514	8	269	15.4%	2.24 [1.02, 4.92]
Takai et al, 2017	37	1339	59	1797	20.9%	0.84 [0.55, 1.27]
**Subtotal (95% CI)**		**2004**		**2176**	**43.3%**	**1.21 [0.54, 2.73]**
Total events	72		69			
Heterogeneity: Tau^2 = 0.32; Chi^2 = 7.70, df = 4 (P = 0.10); I^2 = 48%						
Test for overall effect: Z = 0.46 (P = 0.64)						
**Total (95% CI)**		**4622**		**3256**	**100.0%**	**1.39 [0.84, 2.32]**
Total events	172		95			
Heterogeneity: Tau^2 = 0.28; Chi^2 = 21.81, df = 14 (P = 0.08); I^2 = 36%						
Test for overall effect: Z = 1.27 (P = 0.20)						
Test for subgroup differences: Chi^2 = 0.20, df = 1 (P = 0.65), I^2 = 0%						

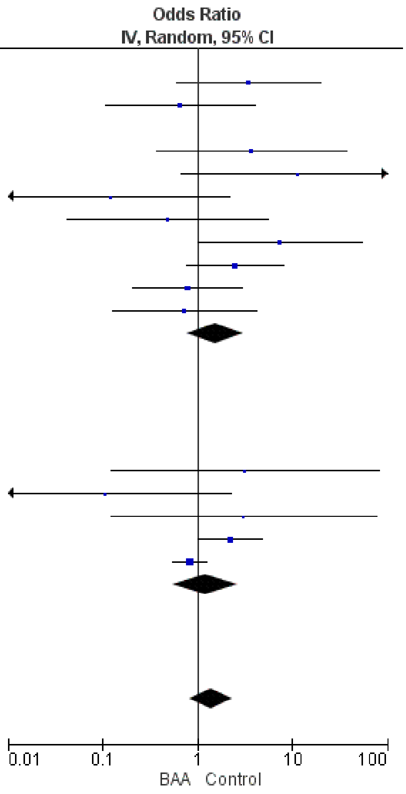

Study or Subgroup	LAA Events	LAA Total	PVI Events	PVI Total	Weight	Odds Ratio IV, Random, 95% CI
Alexander et al, 2018	1	25	1	27	7.9%	1.08 [0.06, 18.30]
McCarthy et al, 2010	7	175	2	50	24.6%	1.00 [0.20, 4.97]
Srivastava et al, 2008	2	40	0	40	6.7%	5.26 [0.24, 113.11]
Stulak et al, 2014	1	110	8	269	14.5%	0.30 [0.04, 2.42]
Takai et al, 2017	3	266	59	1797	46.4%	0.34 [0.10, 1.08]
**Total (95% CI)**		**616**		**2183**	**100.0%**	**0.57 [0.26, 1.26]**
Total events	14		70			
Heterogeneity: Tau^2 = 0.00; Chi^2 = 3.84, df = 4 (P = 0.43); I^2 = 0%						
Test for overall effect: Z = 1.39 (P = 0.17)						

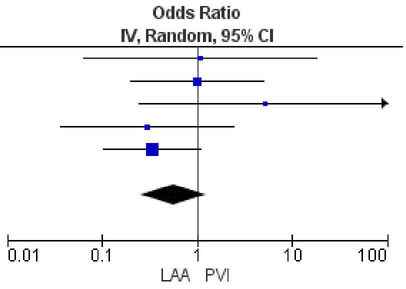

E

Renal Failure

Forest Plot of Renal Failure (Conventional Pairwise Meta-Analysis)

Study or Subgroup	BAA Events	BAA Total	LAA Events	LAA Total	Weight	Odds Ratio IV, Random, 95% CI
**2.1.1 BAA VS LAA**						
Stulak et al, 2014	10	514	3	110	1.2%	0.71 [0.19, 2.61]
Takai et al, 2017	339	1339	58	266	20.0%	1.22 [0.89, 1.67]
Yang et al, 2017	6	94	2	35	0.7%	1.13 [0.22, 5.86]
**Subtotal (95% CI)**		**1947**		**411**	**21.9%**	**1.18 [0.87, 1.59]**
Total events	355		63			
Heterogeneity: Tau^2 = 0.00; Chi^2 = 0.63, df = 2 (P = 0.73); I^2 = 0%						
Test for overall effect: Z = 1.06 (P = 0.29)						
**2.1.2 BAA VS PVI**						
Stulak et al, 2014	10	514	9	269	2.4%	0.57 [0.23, 1.43]
Takai et al, 2017	339	1339	463	1797	75.7%	0.98 [0.83, 1.15]
**Subtotal (95% CI)**		**1853**		**2066**	**78.1%**	**0.91 [0.64, 1.30]**
Total events	349		472			
Heterogeneity: Tau^2 = 0.03; Chi^2 = 1.27, df = 1 (P = 0.26); I^2 = 21%						
Test for overall effect: Z = 0.52 (P = 0.60)						
**Total (95% CI)**		**3800**		**2477**	**100.0%**	**1.00 [0.87, 1.16]**
Total events	704		535			
Heterogeneity: Tau^2 = 0.00; Chi^2 = 3.26, df = 4 (P = 0.52); I^2 = 0%						
Test for overall effect: Z = 0.07 (P = 0.95)						
Test for subgroup differences: Chi^2 = 1.18, df = 1 (P = 0.28), I^2 = 15.2%						

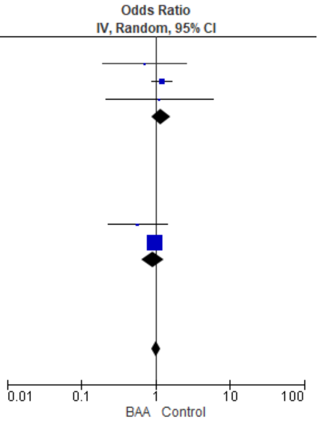

Study or Subgroup	PVI Events	PVI Total	LAA Events	LAA Total	Weight	Odds Ratio IV, Random, 95% CI
Stulak et al, 2014	9	269	3	110	5.2%	1.23 [0.33, 4.65]
Takai et al, 2017	463	1797	58	266	94.8%	1.24 [0.91, 1.70]
**Total (95% CI)**		**2066**		**376**	**100.0%**	**1.24 [0.92, 1.68]**
Total events	472		61			

Heterogeneity: Tau² = 0.00; Chi² = 0.00, df = 1 (P = 0.99); I² = 0%
Test for overall effect: Z = 1.42 (P = 0.16)

Odds Ratio IV, Random, 95% CI

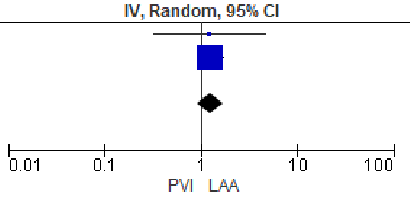

F

Cerebrovascular Accidents

Forest Plot of Cerebrovascular Accidents (Conventional Pairwise Meta-Analysis)

Study or Subgroup	BAA Events	BAA Total	Control Events	Control Total	Weight	Odds Ratio IV, Random, 95% CI
**1.1.1 BAA vs LAA**						
Albage et al, 2011	1	44	2	71	3.8%	0.80 [0.07, 9.12]
Bogachev et al, 2018	3	70	1	70	4.2%	3.09 [0.31, 30.45]
Charitos et al, 2015	1	29	0	22	2.3%	2.37 [0.09, 60.96]
Kim et al, 2011	3	199	14	85	10.1%	0.08 [0.02, 0.28]
McCarthy et al, 2010	2	91	1	175	3.8%	3.91 [0.35, 43.71]
Niv Ad et al, 2017	6	93	9	93	12.3%	0.64 [0.22, 1.89]
Stulak et al, 2014	6	514	0	110	2.8%	2.82 [0.16, 50.52]
Takai et al, 2017	68	1339	16	266	20.4%	0.84 [0.48, 1.47]
Yang et al, 2017	7	94	1	35	4.7%	2.74 [0.32, 23.08]
**Subtotal (95% CI)**		**2473**		**927**	**64.5%**	**0.89 [0.39, 2.05]**
Total events	97		44			
Heterogeneity: Tau² = 0.75; Chi² = 18.33, df = 8 (P = 0.02); I² = 56%						
Test for overall effect: Z = 0.28 (P = 0.78)						
**1.1.2 BAA vs PVI**						
Albrecht et al, 2009	1	20	2	20	3.7%	0.47 [0.04, 5.69]
McCarthy et al, 2010	2	91	0	50	2.5%	2.82 [0.13, 59.92]
Stulak et al, 2014	6	514	1	269	4.8%	3.17 [0.38, 26.43]
Takai et al, 2017	68	1339	108	1797	24.6%	0.84 [0.61, 1.14]
**Subtotal (95% CI)**		**1964**		**2136**	**35.5%**	**0.86 [0.64, 1.17]**
Total events	77		111			
Heterogeneity: Tau² = 0.00; Chi² = 2.28, df = 3 (P = 0.52); I² = 0%						
Test for overall effect: Z = 0.95 (P = 0.34)						
**Total (95% CI)**		**4437**		**3063**	**100.0%**	**0.86 [0.52, 1.44]**
Total events	174		155			

Heterogeneity: Tau² = 0.25; Chi² = 20.94, df = 12 (P = 0.05); I² = 43%
Test for overall effect: Z = 0.57 (P = 0.57)
Test for subgroup differences: Chi² = 0.00, df = 1 (P = 0.95), I² = 0%

Odds Ratio IV, Random, 95% CI

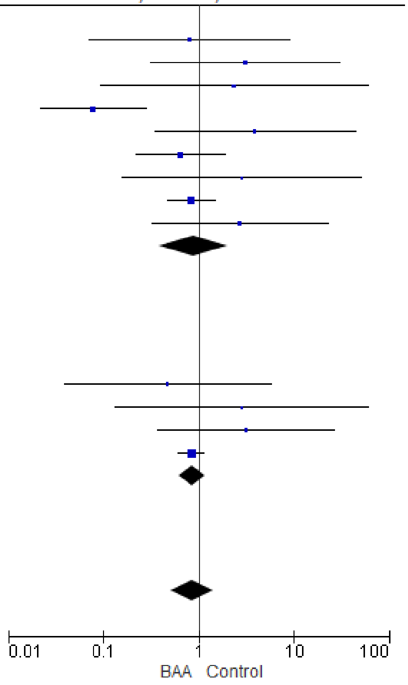

Study or Subgroup	PVI Events	PVI Total	LAA Events	LAA Total	Weight	Odds Ratio IV, Random, 95% CI
McCarthy et al, 2010	0	50	1	175	2.7%	1.15 [0.05, 28.71]
Stulak et al, 2014	1	269	0	110	2.7%	1.23 [0.05, 30.54]
Takai et al, 2017	108	1797	16	266	94.6%	1.00 [0.58, 1.72]
**Total (95% CI)**		**2116**		**551**	**100.0%**	**1.01 [0.60, 1.71]**
Total events	109		17			

Heterogeneity: Tau² = 0.00; Chi² = 0.02, df = 2 (P = 0.99); I² = 0%
Test for overall effect: Z = 0.03 (P = 0.97)

Odds Ratio IV, Random, 95% CI

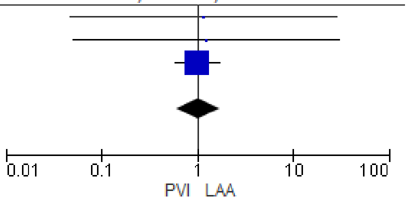

G

All-cause Mortality

Forest Plot of All-cause Mortality (Conventional Pairwise Meta-Analysis)

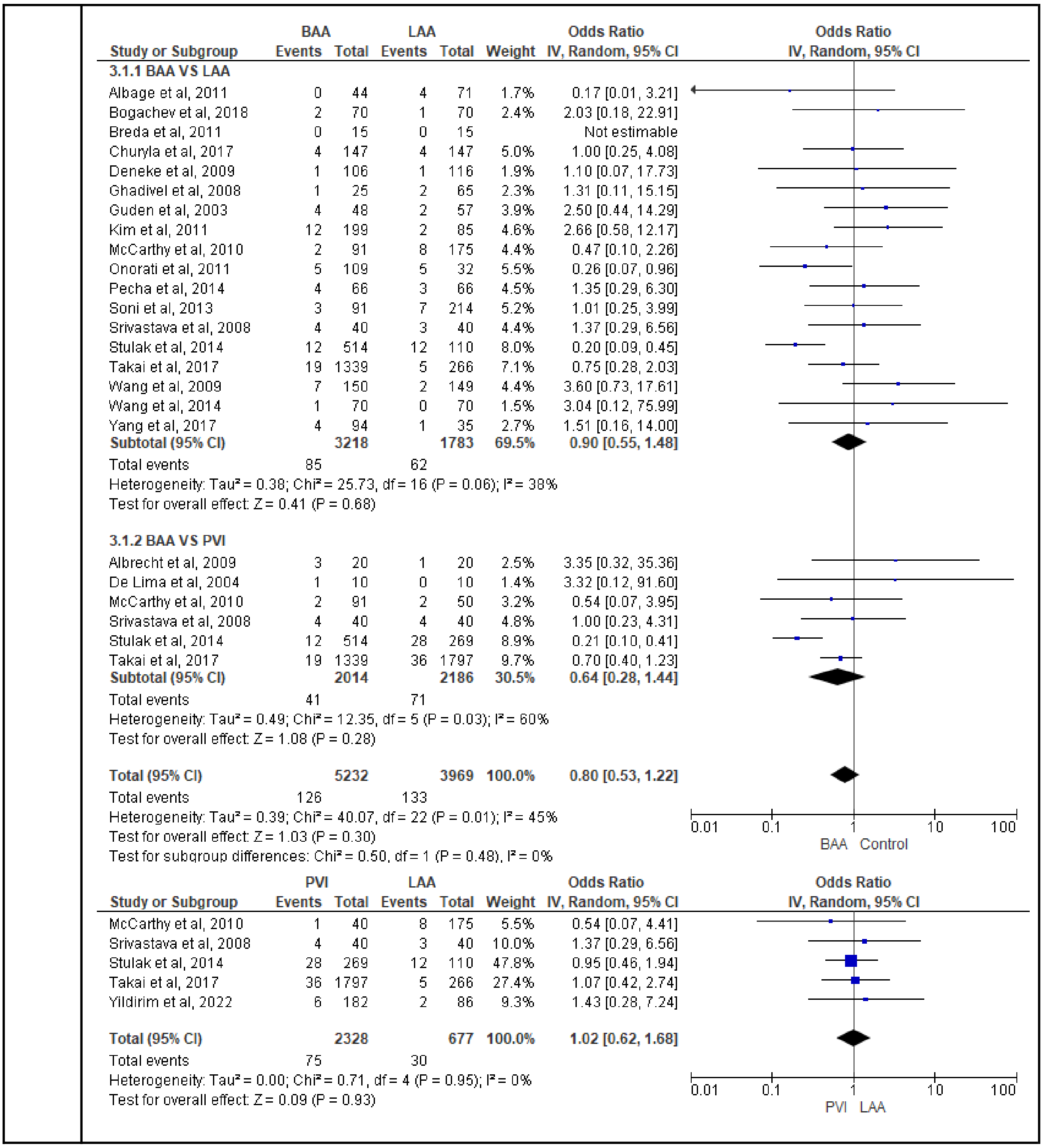

Overall postoperative outcomes were reported for patients who underwent ablation surgery. (A) Forest plot for non-sinus rhythm demonstrating low until moderate heterogeneity; (B) Forest plot for pacemaker implantation demonstrating insignificant heterogeneity (E) Forest plot for renal failure demonstrating insignificant heterogeneity; (F) Forest plot for cerebrovascular accidents demonstrating insignificant until low heterogeneity; (G) Forest plot for all caused mortality demonstrating insignificant until low heterogeneity

Figure S2. Overall operative procedure

A — **Cross-clamp (Xc) time**

Forest Plot of Xc Time (Conventional Pairwise Meta-Analysis)

Study or Subgroup	BAA Events	BAA Total	Control Events	Control Total	Weight	Odds Ratio IV, Random, 95% CI
**1.1.1 BAA vs LAA**						
Albage et al, 2011	5	44	6	71	4.0%	1.39 [0.40, 4.85]
Bogachev et al, 2018	8	70	4	70	4.0%	2.13 [0.61, 7.43]
Churyla et al, 2017	19	147	10	147	9.7%	2.03 [0.91, 4.54]
Gauzebroek et al, 2008	16	25	8	28	4.7%	4.44 [1.40, 14.14]
Guden et al, 2003	12	48	10	57	7.0%	1.57 [0.61, 4.03]
Kalybekova et al, 2021	6	58	3	58	3.0%	2.12 [0.50, 8.90]
Kim et al, 2011	3	199	0	85	0.7%	3.05 [0.16, 59.61]
Pecha et al, 2014 (A)	15	131	26	463	14.0%	2.17 [1.11, 4.24]
Pecha et al, 2014 (B)	7	66	4	66	3.8%	1.84 [0.51, 6.61]
Soni et al, 2013	15	91	16	214	11.1%	2.44 [1.15, 5.18]
Srivastava et al, 2008	0	40	1	40	0.6%	0.33 [0.01, 8.22]
Stulak et al, 2014	12	514	3	110	3.8%	0.85 [0.24, 3.07]
Takami et al, 2019	2	30	1	20	1.0%	1.36 [0.11, 16.05]
Wang et al, 2009	2	150	1	149	1.1%	2.00 [0.18, 22.30]
Wang et al, 2014	3	70	2	70	1.9%	1.52 [0.25, 9.40]
**Subtotal (95% CI)**		**1683**		**1648**	**70.4%**	**1.98 [1.47, 2.67]**
Total events	125		95			
Heterogeneity: Tau² = 0.00; Chi² = 5.94, df = 14 (P = 0.97); I² = 0%						
Test for overall effect: Z = 4.49 (P < 0.00001)						
**1.1.2 BAA vs PVI**						
De Lima et al, 2004	0	10	0	10		Not estimable
DeRose, Jr et al, 2019	16	64	10	62	8.0%	1.73 [0.72, 4.19]
Gauzebroek et al, 2008	16	25	2	12	2.1%	8.89 [1.59, 49.83]
Hald et al, 2021	13	109	20	337	11.6%	2.15 [1.03, 4.47]
Srivastava et al, 2008	0	40	0	40		Not estimable
Stulak et al, 2014	12	514	7	269	7.0%	0.89 [0.35, 2.30]
Takasaki et al, 2012	1	35	1	40	0.8%	1.15 [0.07, 19.05]
**Subtotal (95% CI)**		**797**		**770**	**29.6%**	**1.83 [1.01, 3.31]**
Total events	58		40			
Heterogeneity: Tau² = 0.13; Chi² = 5.73, df = 4 (P = 0.22); I² = 30%						
Test for overall effect: Z = 1.99 (P = 0.05)						
**Total (95% CI)**		**2480**		**2418**	**100.0%**	**1.92 [1.50, 2.47]**
Total events	183		135			
Heterogeneity: Tau² = 0.00; Chi² = 11.80, df = 19 (P = 0.89); I² = 0%						
Test for overall effect: Z = 5.12 (P < 0.00001)						
Test for subgroup differences: Chi² = 0.05, df = 1 (P = 0.82), I² = 0%						

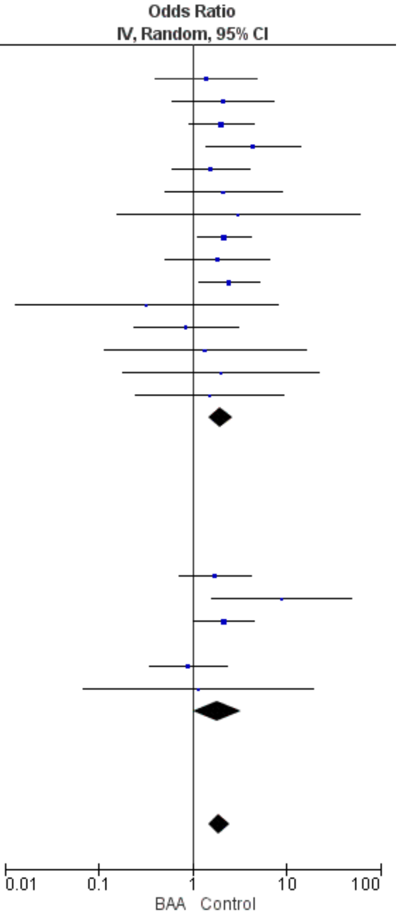

Study or Subgroup	LAA Mean	LAA SD	LAA Total	PVI Mean	PVI SD	PVI Total	Weight	Mean Difference IV, Random, 95% CI
Alexander et al, 2018	78.7	16.5	25	63.2	12.3	27	2.0%	15.50 [7.54, 23.46]
Gauzebroek et al, 2008	83	34	28	68	28	12	0.3%	15.00 [-5.24, 35.24]
Stulak et al, 2014	81	6	110	65	6	269	70.0%	16.00 [14.67, 17.33]
Takai et al, 2017	127	52	266	109	47	1797	2.8%	18.00 [11.38, 24.62]
Yildirim et al, 2022	89	9	86	71	8	182	24.9%	18.00 [15.77, 20.23]
**Total (95% CI)**			**515**			**2287**	**100.0%**	**16.54 [15.43, 17.66]**
Heterogeneity: Tau² = 0.00; Chi² = 2.56, df = 4 (P = 0.63); I² = 0%								
Test for overall effect: Z = 29.12 (P < 0.00001)								

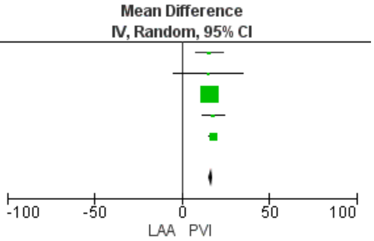

B — **Cardiopulmonary bypass (CPB) time**

Forest Plot of CPB Time (Conventional Pairwise Meta-Analysis)

Study or Subgroup	BAA Mean	BAA SD	BAA Total	Control Mean	Control SD	Control Total	Weight	Mean Difference IV, Random, 95% CI
**5.1.1 BAA vs LAA**								
Breda et al, 2011	100	23.52	15	50.65	15.17	15	4.8%	49.35 [35.19, 63.51]
Calo et al, 2006	228	32	39	164	25	41	5.0%	64.00 [51.37, 76.63]
Deneke et al, 2009	149	36	64	135	28	66	5.2%	14.00 [2.89, 25.11]
Gauzebroek et al, 2008	147	49	25	126	42	28	3.4%	21.00 [-3.72, 45.72]
Ghadivel et al, 2008	141	40	25	120	37	65	4.3%	21.00 [2.92, 39.08]
Kim et al, 2011	171.3	57.4	199	165.6	46.3	85	5.0%	5.70 [-6.97, 18.37]
Soni et al, 2013	152	34	91	136	54	214	5.3%	16.00 [5.94, 26.06]
Stulak et al, 2014	103	7	514	109	7	110	6.0%	-6.00 [-7.44, -4.56]
Takai et al, 2017	190	67	1339	187	80	266	5.3%	3.00 [-7.26, 13.26]
Takami et al, 2019	248.2	59	30	200.1	25.7	20	3.5%	48.10 [24.17, 72.03]
Wang et al, 2009	138.2	46	70	101	34	70	4.9%	37.20 [23.80, 50.60]
Wang et al, 2014	128	40	150	114	41	149	5.4%	14.00 [4.82, 23.18]
Yang et al, 2017	151.8	45	94	145.4	50	35	4.2%	6.40 [-12.50, 25.30]
Yoshikawa et al, 2006	187.5	44.6	29	187	48.6	71	4.1%	0.50 [-19.28, 20.28]
**Subtotal (95% CI)**			**2684**			**1235**	**66.6%**	**20.65 [8.22, 33.07]**
Heterogeneity: Tau² = 503.74; Chi² = 279.06, df = 13 (P < 0.00001); I² = 95%								
Test for overall effect: Z = 3.26 (P = 0.001)								
**5.1.2 BAA vs PVI**								
Albrecht et al, 2009	122.95	21	20	99.85	23.8	20	4.9%	23.10 [9.19, 37.01]
De Lima et al, 2004	115.3	25	10	97.8	3	10	4.6%	17.50 [1.89, 33.11]
Gauzebroek et al, 2008	147	49	25	102	28	12	3.4%	45.00 [20.10, 69.90]
Stulak et al, 2014	104	7	514	90	8	269	6.0%	14.00 [12.87, 15.13]
Takai et al, 2017	190	67	1339	171	71	1797	5.8%	19.00 [14.14, 23.86]
Takasaki et al, 2012	143	37	35	138	42	40	4.3%	5.00 [-12.88, 22.88]
Wynn et al, 2016	209	52	63	172	44	51	4.3%	37.00 [19.37, 54.63]
**Subtotal (95% CI)**			**2006**			**2199**	**33.4%**	**19.39 [13.43, 25.35]**
Heterogeneity: Tau² = 29.47; Chi² = 18.75, df = 6 (P = 0.005); I² = 68%								
Test for overall effect: Z = 6.38 (P < 0.00001)								
**Total (95% CI)**			**4690**			**3434**	**100.0%**	**20.71 [13.72, 27.69]**
Heterogeneity: Tau² = 211.11; Chi² = 661.89, df = 20 (P < 0.00001); I² = 97%								
Test for overall effect: Z = 5.81 (P < 0.00001)								
Test for subgroup differences: Chi² = 0.03, df = 1 (P = 0.86), I² = 0%								

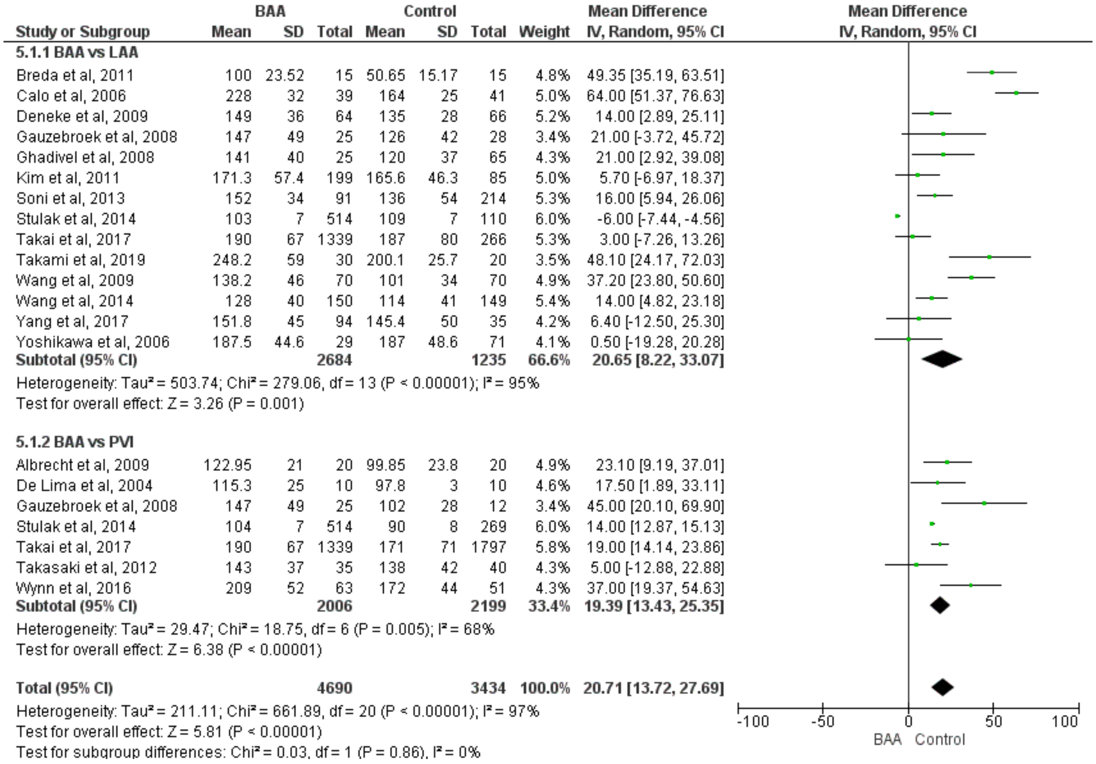

Study or Subgroup	LAA Mean	LAA SD	LAA Total	PVI Mean	PVI SD	PVI Total	Weight	Mean Difference IV, Random, 95% CI
Alexander et al, 2018	99.2	13.4	25	79.7	10.1	27	5.7%	19.50 [13.01, 25.99]
Gauzebroek et al, 2008	126	42	28	102	28	12	0.5%	24.00 [1.80, 46.20]
Stulak et al, 2014	109	7	110	90	8	269	91.5%	19.00 [17.38, 20.62]
Takai et al, 2017	187	80	266	171	71	1797	2.3%	16.00 [5.84, 26.16]
**Total (95% CI)**			**429**			**2105**	**100.0%**	**18.98 [17.43, 20.53]**
Heterogeneity: Tau² = 0.00; Chi² = 0.55, df = 3 (P = 0.91); I² = 0%								
Test for overall effect: Z = 24.01 (P < 0.00001)								

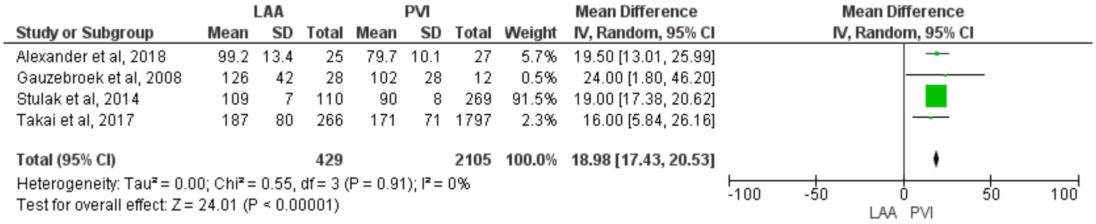

C

Ablation Time

Forest Plot of Ablation Time (Conventional Pairwise Meta-Analysis)

Study or Subgroup	BAA Mean	BAA SD	BAA Total	Control Mean	Control SD	Control Total	Weight	Mean Difference IV, Random, 95% CI
**7.1.1 BAA vs LAA**								
Breda et al, 2011	7	0.63	15	4	0.78	15	17.7%	3.00 [2.49, 3.51]
Calo et al, 2006	41	14	39	31	1	41	13.5%	10.00 [5.60, 14.40]
Ghadivel et al, 2008	13	5.1	25	10	3.5	65	16.5%	3.00 [0.83, 5.17]
Srivastava et al, 2008	17.75	4.32	40	7.1	1.57	40	17.2%	10.65 [9.23, 12.07]
Wang et al, 2009	29	2	150	21	3	149	17.6%	8.00 [7.42, 8.58]
**Subtotal (95% CI)**			**269**			**310**	**82.4%**	**6.79 [3.50, 10.08]**
Heterogeneity: Tau² = 12.91; Chi² = 224.35, df = 4 (P < 0.00001); I² = 98%								
Test for overall effect: Z = 4.04 (P < 0.0001)								
**7.1.2 BAA vs PVI**								
Srivastava et al, 2008	17.75	4.32	40	5	1.71	40	17.1%	12.75 [11.31, 14.19]
Wynn et al, 2016	72.5	188	63	41.7	14.6	61	0.5%	30.80 [-15.77, 77.37]
**Subtotal (95% CI)**			**103**			**101**	**17.6%**	**12.77 [11.33, 14.21]**
Heterogeneity: Tau² = 0.00; Chi² = 0.58, df = 1 (P = 0.45); I² = 0%								
Test for overall effect: Z = 17.39 (P < 0.00001)								
**Total (95% CI)**			**372**			**411**	**100.0%**	**7.94 [4.65, 11.24]**
Heterogeneity: Tau² = 15.92; Chi² = 317.04, df = 6 (P < 0.00001); I² = 98%								
Test for overall effect: Z = 4.73 (P < 0.00001)								
Test for subgroup differences: Chi² = 10.65, df = 1 (P = 0.001), I² = 90.6%								

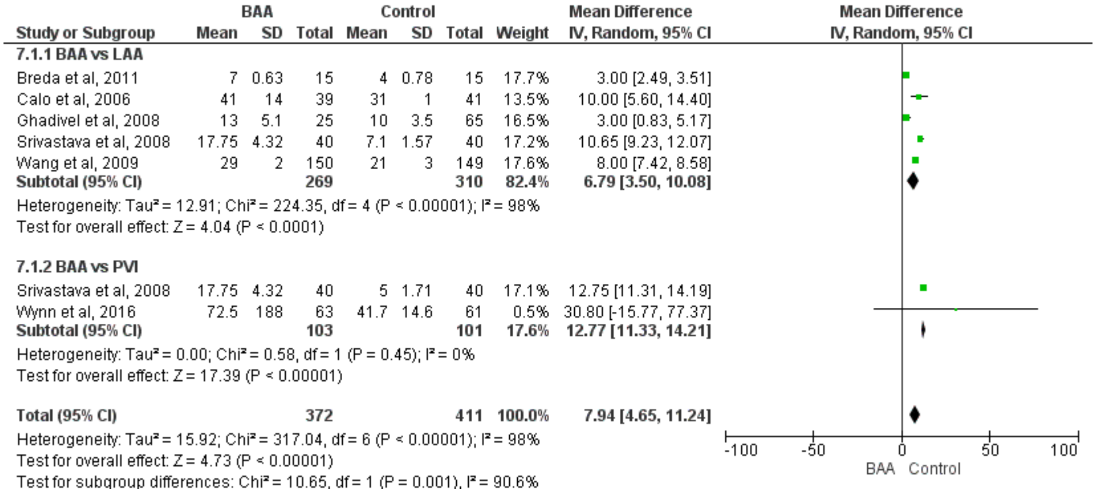

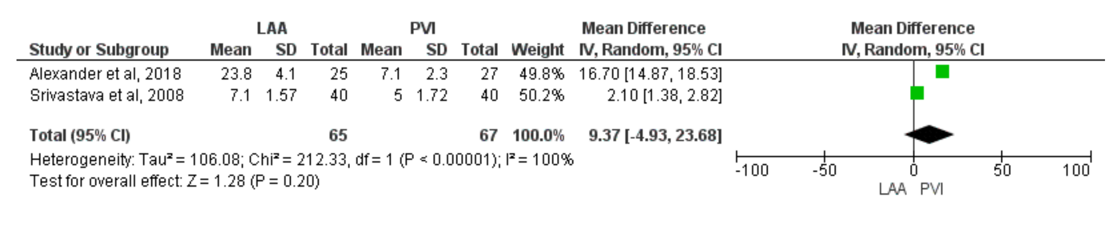

Overall operative procedure time was reported for patients who underwent ablation surgery. (A) Forest plot for cross-clamp (Xc) time demonstrating insignificant heterogeneity; (B) Forest plot for cardiopulmonary bypass (CPB) time demonstrating insignificant to high heterogeneity; (C) Forest plot for the ablation time demonstrating high heterogeneity.

Figure S3. Hospital admission and stay

A — Hospital Admissions

Forest Plot of Hospital Admissions (Conventional Pairwise Meta-Analysis)

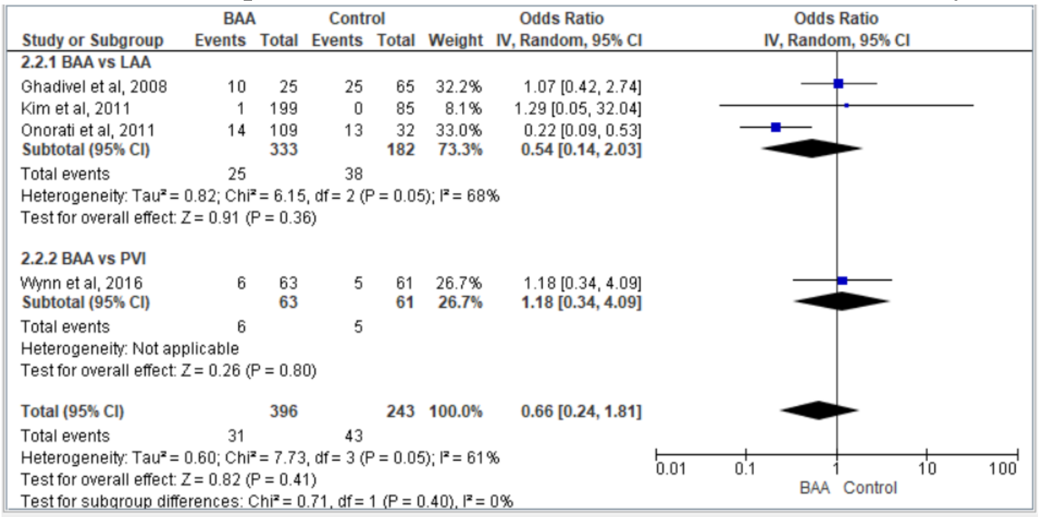

B — Total Length of Stay

Forest Plot of Length of Stay (Conventional Pairwise Meta-Analysis)

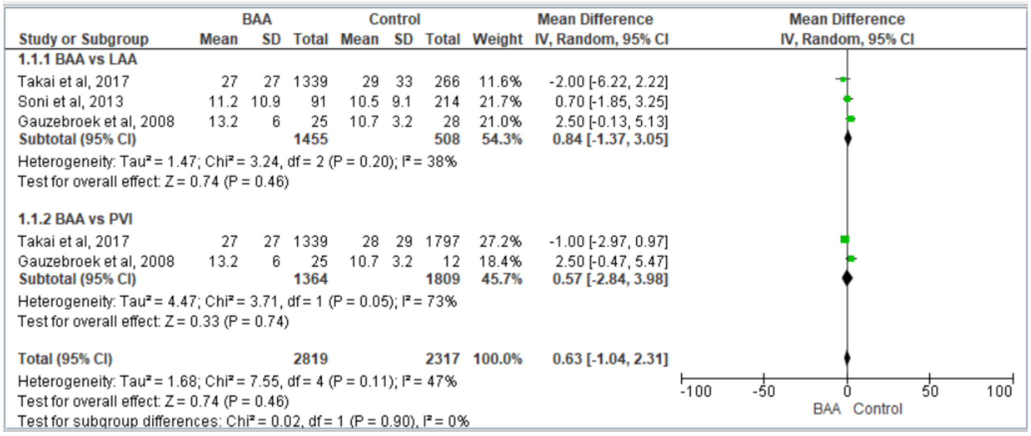

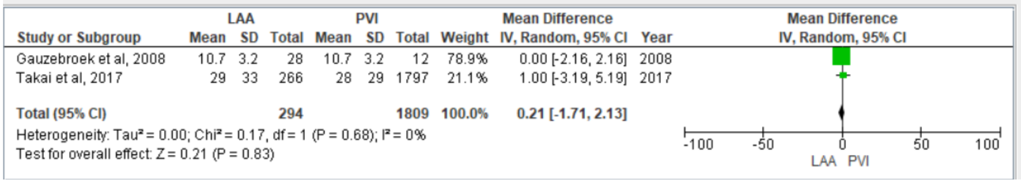

Overall hospital stay reported for patients who underwent MVr/R surgery.

(A) Forest plot for hospital admissions demonstrating insignificant heterogeneity; (B) Forest plot for the total length of stay in the hospital demonstrating low until insignificant heterogeneity.

Table S6. Critical Appraisal

Clinical Trials

		Domain 1a. Randomization process				: Risk of bias arising from the timing of identification or recruitment of pa				Domain 2. Deviations from intended interventions									Domain 3. Mising outcome data						Domain 4. Measurement of the outcome							Domain 5. Selection of the reported result				Domain 6. Overall Bias
No	Author (year)	1a.1	1a.2	1a.3	Risk-of-bias judge	1b.1	1b.2	1b.3	Risk-of-bias judge	2.1a	2.1b (2.1)	2.2	2.3	2.4	2.5	2.6	2.7	Risk-of-bias judge	3.1a	3.1b	3.2	3.3	3.4	Risk-of-bias judge	4.1	4.2	4.3a	4.3b	4.4	4.5	Risk-of-bias judge	5.1	5.2	5.3	Risk-of-bias judge	Risk-of-bias judgement
1	Hald et al, 2021																																			
2	Albrecht et al, 2009	Y	Y	N	Low	Y	N	PN	Low	N	PN	Y	N	N	N	Y	N	Low	Y	Y	PY	PN	PN	Low	N	Y	Y	Y	PN	PN	Low	Y	N	PN	Low	Low
3	Srivastava et al, 2008	Y	Y	N	Low	Y	N	PN	Low	N	PN	Y	N	N	N	Y	N	Low	Y	Y	PY	PN	PN	Low	N	Y	Y	Y	PN	PN	Low	Y	N	PN	Low	Low
4	Stulak et al, 2014																																			
5	Onorati et al, 2011																																			
6	Alexander et al, 2018	Y	Y	N	Low	Y	NA	N	Low	PN	NA	PN	NA	NA	NA	Y	NA	Low	Y	Y	NA	NA	NA	Low	N	N	N	NA	NA	NA	Low	Y	PN	PN	Low	Low
7	Gauzebroek et al, 2008																																			
8	Ghadivel et al, 2008	N	N	NI	High	N	PN	PY	Some Concerns	N	NA	PN	NA	NA	NA	Y	NA	Low	Y	PY	NA	NA	NA	Low	NI	NI	PN	NA	NA	NA	Some Concerns	PY	PN	PN	Low	High
9	Gillinov et al, 2015	Y	Y	N	Low	Y	NA	N	Low	N	NA	N	NA	NA	NA	Y	NA	Low	Y	Y	NA	NA	NA	Low	N	N	N	NA	NA	NA	Low	Y	N	N	Low	Low
10	Yildirim et al, 2022																																			
11	Wynn et al, 2016	Y	Y	N	Low	Y	NA	N	Low	N	NA	N	NA	NA	NA	Y	NA	Low	Y	Y	NA	NA	NA	Low	N	N	N	NA	NA	NA	Low	Y	PN	PN	Low	Low
12	Wang et al, 2009	Y	Y	N	Low	Y	NA	N	Low	N	NA	PN	NA	NA	NA	Y	NA	Low	Y	Y	NA	NA	NA	Low	N	N	PN	NA	NA	NA	Low	Y	PN	PN	Low	Low
13	Calo et al, 2006	Y	Y	N	Low	Y	NA	N	Low	N	NA	PN	NA	NA	NA	Y	NA	Low	Y	Y	NA	NA	NA	Low	N	N	PN	NA	NA	NA	Low	Y	PN	PN	Low	Low
14	Henn et al, 2015																																			
15	Albage et al, 2011																																			
16	McCarthy et al, 2010																																			
17	Guden et al, 2003																																			
18	Pecha et al, 2014																																			
19	Gualis et al, 2016																																			
20	Deneke et al, 2009																																			
21	Pecha et al, 2014																																			
22	Soni et al, 2013																																			
23	Kim et al, 2011																																			
24	Breda et al, 2011																																			
25	Deneke et al, 2007	Y	Y	N	Low	Y	N	PN	Low	N	PN	Y	N	N	N	Y	N	Low	Y	Y	PY	PN	PN	Low	N	Y	Y	Y	PN	PN	Low	Y	N	PN	Low	Low
26	Ryan et al, 2004																																			
27	Takami et al, 2019																																			
28	Takasaki et al, 2012																																			
29	Charitos et al, 2015																																			
30	Churyla et al, 2017																																			
31	Niv Ad et al, 2017	Y	Y	N	Low	Y	N	PY	Low	Y	PN	Y	N	N	N	Y	N	Low	Y	Y	PY	PN	PN	Low	N	N	Y	Y	PN	PN	Low	Y	PN	PN	Low	Low
32	Yang et al, 2017																																			
33	Takai et al, 2017																																			
34	Bogachev et al, 2018	Y	Y	N	Low	Y	N	PN	Low	N	PN	Y	N	N	N	Y	N	Low	Y	Y	PY	PN	PN	Low	N	Y	Y	Y	PN	PN	Low	Y	N	PN	Low	Low
35	Yoshikawa et al, 2006																																			
36	Kalybekova et al, 2021																																			
37	DeRose, Jr et al, 2019																																			
38	De Lima et al, 2004	Y	Y	N	Low	Y	N	N	Low	N	PN	Y	N	N	N	Y	N	Low	Y	Y	PY	PN	PN	Low	N	Y	Y	Y	PN	N	Low	Y	N	PN	Low	Low
39	Wang et al, 2014																																			

Observational studies

		Selection: Representativeness of the exposed cohort				Selection of the non exposed cohort			Ascertainment of exposure				Demonstration that outcome of interest was not prese		Total stars	Comparability: Comparability of cohorts on the bas		Total stars	Outcome: Assessment of outcome				Was follow-up long enough for outc		Adequacy of follow up of cohorts				Total stars	Total stars for overall sections	Quality Assessment based on AHRQ
No	Author (year)	Truly representative of the average patients that received BAA and/or LAA and/ir PVI in the community *	Somewhat representative of the average patients that received BAA and/or LAA and/ir PVI in the community *	Selected group of users eg nurses, volunteers	No description of the derivation of the cohort	Drawn from the same community as the exposed cohort *	Drawn from a different source	No description of the derivation of the non exposed cohort	Secure record (eg medical records) *	Structured interview *	Written self report	No description	Yes *	No		Study controls for patients that underwent concomitant surgical ablation (BAA and/or LAA and/or PVI)*	Study controls for any additional factor * (This criteria could be modified to indicate specific control for a second important factor.)		Independent blind	Record linkage *	Self-report	No description	Yes (1 month of th	No	Complete follow u	Subjects lost to f	Follow up rate <	No statement			
1	Hald et al, 2021	v				v			v				v		4	v		1		v			v		v				3	8	Good quality
2	Albrecht et al, 2009																														
3	Srivastava et al, 2008																														
4	Stulak et al, 2014	v				v			v				v		4	v		1		v			v		v				3	8	Good quality
5	Onorati et al, 2011		v			v			v				v		4	v	v	2		v			v		v				3	9	Good quality
6	Alexander et al, 2018																														
7	Gauzebroek et al, 2008		v			v			v				v		4	v	v	2		v			v			v			3	9	Good quality
8	Ghadivel et al, 2008																														
9	Gillinov et al, 2015																														
10	Yildirim et al, 2022		v			v			v				v		4	v	v	2		v			v					v	2	8	Good quality
11	Wynn et al, 2016																														
12	Wang et al, 2009																														
13	Calo et al, 2006																														
14	Henn et al, 2015		v			v			v				v		4	v	v	2		v			v				v		2	8	Good quality
15	Albage et al, 2011		v			v			v				v		4	v	v	2		v			v			v			3	9	Good quality
16	McCarthy et al, 2010		v			v			v					v	3	v	v	2		v			v			v			3	8	Good quality
17	Guden et al, 2003		v			v			v					v	3	v	v	2		v			v				v		2	8	Good quality
18	Pecha et al, 2014		v			v			v				v		4	v	v	2		v			v					v	2	7	Good quality
19	Gualis et al, 2016		v			v			v					v	3	v	v	2		v			v					v	2	8	Good quality
20	Deneke et al, 2009		v			v			v					v	3	v	v	2		v			v				v		2	7	Good quality
21	Pecha et al, 2014		v			v			v				v		4	v	v	2		v			v		v				3	9	Good quality
22	Soni et al, 2013		v			v			v				v		4	v	v	2		v			v		v				3	9	Good quality
23	Kim et al, 2011		v			v			v					v	3	v	v	2	v				v			v			3	8	Good quality
24	Breda et al, 2011		v			v			v					v	3	v	v	2	v				v		v				3	8	Good quality
25	Deneke et al, 2007																														
26	Ryan et al, 2004		v			v			v				v		4	v	v	2	v				v			v			3	9	Good quality
27	Takami et al, 2019		v			v			v				v		4	v	v	2	v				v				v		2	8	Good quality
28	Takasaki et al, 2012		v			v			v				v		4	v	v	2	v				v		v				3	9	Good quality
29	Charitos et al, 2015		v			v			v					v	3	v	v	2	v				v		v				3	8	Good quality
30	Churyla et al, 2017		v			v			v					v	3	v	v	2	v				v			v			3	8	Good quality
31	Niv Ad et al, 2017																														
32	Yang et al, 2017		v			v			v				v		4	v	v	2	v				v			v			3	9	Good quality
33	Takai et al, 2017		v			v			v				v		4	v	v	2	v				v		v				3	9	Good quality
34	Bogachev et al, 2018																														
35	Yoshikawa et al, 2006		v			v			v				v		4	v	v	2	v				v		v				3	9	Good quality
36	Kalybekova et al, 2021		v			v			v				v		4	v	v	2	v				v		v				3	9	Good quality
37	DeRose, Jr et al, 2019		v			v			v				v		4	v	v	2	v				v			v			3	9	Good quality
38	De Lima et al, 2004																														
39	Wang et al, 2014		v			v			v				v		4	v	v	2	v				v		v				3	9	Good quality
